# StretchView – A Multi‐Axial Cell‐Stretching Device for Long‐Term Automated Videomicroscopy of Living Cells

**DOI:** 10.1002/advs.202408853

**Published:** 2025-01-10

**Authors:** David Jaworski, Lara Hundsdorfer, Effie Bastounis, Iordania Constantinou

**Affiliations:** ^1^ Institute of Microtechnology (IMT) Technische Universität Braunschweig Alte Salzdahlumer Str. 203 38124 Braunschweig Germany; ^2^ Center of Pharmaceutical Engineering (PVZ) Technische Universität Braunschweig Franz‐Liszt‐Str. 35a 38106 Braunschweig Germany; ^3^ Interfaculty Institute of Microbiology and Infection Medicine (IMIT) University of Tübingen Auf der Morgenstelle 28 72076 Tübingen Germany; ^4^ Cluster of Excellence “Controlling Microbes to Fight Infections” (CMFI), EXC 2124 University of Tübingen Auf der Morgenstelle 28 72076 Tübingen Germany

**Keywords:** cell stretching device, digital image correlation, elastomeric device, live‐cell imaging, mechanobiology

## Abstract

Incorporating mechanical stretching of cells in tissue culture is crucial for mimicking (patho)‐physiological conditions and understanding the mechanobiological responses of cells, which can have significant implications in areas like tissue engineering and regenerative medicine. Despite the growing interest, most available cell‐stretching devices are not compatible with automated live‐cell imaging, indispensable for characterizing alterations in the dynamics of various important cellular processes. In this work, StretchView is presented, a multi‐axial cell‐stretching platform compatible with automated, time‐resolved live‐cell imaging. Using StretchView, long‐term image acquisition of cells in the relaxed and stretched states is shown for the first time (experimental time of 12 h) without the need for human intervention. Homogeneous and stable strain fields are demonstrated for 18 h of cyclic stretching, highlighting the platform's versatility and robustness. As proof‐of‐principle, the effect of stretching on cell kinematics and spatiotemporal localization of the cell‐cell adhesion protein E‐cadherin is examined for MDCK cells in monolayer. First evidence of a monotonic increase in junctional E‐cadherin localization upon exposure to stretch is presented using live‐cell imaging data acquired during cyclic stretching, suggestive of an increase in barrier integrity of the monolayer. These findings highlight the potential of StretchView in providing insights into cell mechanobiology and beyond.

## Graphical abstract

StretchView is presented, a multi‐axial cell‐stretching platform compatible with automated live‐cell imaging during cyclic stretching. Using StretchView, long‐term image acquisition of epithelial cells and fluorescent tracer beads in relaxed and stretched cell culture membrane states is shown. These capabilities position StretchView as a valuable tool for gaining insights into the dynamics of biomechanically regulated cellular processes.

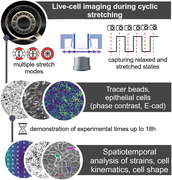

## 1 Introduction

In vivo, cells are continuously subjected to various forms of mechanical stimulation that can influence various cell processes, functions, and ultimately the cell fate. These mechanical forces, whether shear stress from fluid flow over the cells’ apex, compressive or tensile forces applied by the surrounding tissue or cells, are sensed by cells (mechanosensation) and converted into biochemical signals via a process known as mechanotransduction. Mechanotransduction allows cells to probe their mechanical microenvironment and integrate this information, ultimately converting it into complex biochemical signaling responses that play critical roles in a variety of biological processes.^[^ ^1^ ^]^ For example, in the intestine, mechanical stretching can reprogram cells altering their gene expression,^[^ ^2^ ^]^ 3D‐architecture,^[^ ^3^ ^]^ and satiety signals.^[^ ^4^ ^]^ In addition, co‐cultures of cells with bacteria suggest that the gut microbiome is also mechanically regulated,^[^ ^3^, ^5^ ^]^ and that a variety of pathologies, like irritable bowel syndrome are associated with obstruction of proper mechanical stimulation (peristaltic stretching) of cells.^[^ ^6^, ^7^ ^]^ In the eye, cyclic mechanical stretching of trabecular meshwork cells influences extracellular matrix remodeling and steroid biosynthesis, both of which are relevant for intraocular pressure regulation and the pathology of glaucoma.^[^ ^8^, ^9^ ^]^ In blood vessels, mechanical stretch critically impacts the proliferation and barrier integrity of endothelial cells,^[^ ^10^, ^11^ ^]^ while mechanically priming them via stretching in vitro improves the strength and patency of tissue‐engineered vascular implants.^[^ ^12^ ^]^ It is therefore evident that to approximate in vivo conditions in vitro using cell culture‐based systems, it is crucial to carefully consider and incorporate mechanical cues, like stretching, when present in the respective tissue microenvironment.

Although devices to expose cells to fluid flow are commonly used to study the effect of shear stresses on cell biomechanics,^[^ ^13^, ^14^ ^]^ and cell culture on substrates of controlled stiffness is common practice in the field of mechanobiology,^[^ ^15^, ^16^ ^]^ cell‐stretching devices for the application of strain onto cells cultured on elastomeric membranes have been grossly underutilized despite some commercially available options.^[^ ^17^, ^18^, ^19^, ^20^, ^21^ ^]^ This is in part due to the incompatibility of most cell‐stretching systems with microscopy, especially when it comes to long‐term imaging of living cells during stretching. Despite a growing number of cell‐stretching devices being presented in the scientific and patent literature,^[^ ^22^, ^23^, ^24^, ^25^, ^26^, ^27^, ^28^, ^29^, ^30^, ^31^, ^32^, ^33^, ^34^ ^]^ their compatibility with long‐term high‐resolution live‐cell microscopy has either been limited or was not demonstrated or discussed.^[^ ^35^ ^]^

The vast majority of published live‐cell imaging data of stretched and stretching cells is limited to low temporal resolutions or to short experimental times.^[^ ^22^, ^33^, ^34^, ^36^, ^37^ ^]^ For example, Wang et al. reported imaging during cyclic stretching for an experimental time of 3 h at a temporal resolution of 20 min.^[^ ^33^ ^]^ Other studies are limited to static stretch or to imaging after cyclic stretching.^[^ ^26^, ^38^ ^]^ One barrier to high‐resolution live‐cell imaging during stretching is the need for manual refocusing due to the out‐of‐focus motion of the membrane upon stretching.^[^ ^22^, ^26^ ^]^ Manual refocusing can be restricting when experiments require a high stretching frequency or when cyclic cell‐stretching over long experimental periods is desired. Another limitation of existing cell‐stretching devices, especially ones that rely on suspended stretching membranes,^[^ ^39^ ^]^ is the incompatibility with the short working distances of inverted microscope objectives.

Depending on organ function and health status, stretch‐induced strain in tissues and constituent cells can vary in magnitude and direction (uniaxial, biaxial, equibiaxial). Most published and commercial cell‐stretching devices are designed to stretch either uniaxially or (equi‐)biaxially. While this is valuable when simulating (patho)‐physiological conditions, it can be restricting when it comes to the study of the effect or contribution of stretch in fundamental biological processes. For example, the direction of stretch can be crucial in guiding the differentiation of skeletal myocytes (uniaxial stretching drives the differentiation, but equibiaxial stretching does not) which in turn is critical for *ex vivo* tissue engineering.^[^ ^40^ ^]^ It has also been observed that epithelial cell colonies change their morphology perpendicularly upon cyclic uniaxial stretch with a frequency of 0.1 Hz.^[^ ^41^ ^]^ Although there are a few cell‐stretching devices that allow for different axial stretching configurations,^[^ ^22^, ^24^, ^36^, ^42^ ^]^ their design does not usually allow automated switching between uniaxial, biaxial, and equibiaxial stretching within one experiment, with the exception of a recently published cell stretching device with four vacuum chambers.^[^ ^33^ ^]^

In this work, we address the aforementioned limitations of cell‐stretching devices and introduce a novel pneumatic cell‐stretching device that is compatible with live‐cell automated videomicroscopy and also allows switching between stretching axes within single experimental recordings. The novel characteristics of our cell‐stretching devices include enhanced focus stability for cell imaging in the stretched and unstretched configurations in combination with automated long‐term image acquisition and high temporal resolution, all of which are key requirements for increasing the data yield of live‐cell imaging experiments. This aligns with the requirements of live cell imaging in its conventional context, where automated live cell imaging at regular intervals is standard practice. Our cell‐stretching device, referred to as “StretchView”, was used to stretch Madin‐Darby canine kidney (MDCK) cells in a monolayer used as an epithelial cell model. We demonstrate for the first time long‐term (18 h) and automated imaging of a cell‐stretching device membrane in the relaxed and stretched state without manual refocusing. The strain exerted on the cell culture membrane was monitored during the experiments by tracking the displacements of fluorescent tracer particles attached to the membrane and subsequently performing digital image correlation (DIC). As a proof‐of‐principle, we utilized MDCK cells in monolayer and explored alterations in their kinematics and cell shape morphometrics, as well as in the localization of the cell‐cell adhesion protein E‐cadherin during cyclic stretching and as compared to stationary conditions. We present live‐cell imaging data acquired during cyclic cell stretching with a previously unattained combination of long experimental time and high temporal resolution (12 h with intervals of 50 s). Notably, we observed that cyclic stretching of MDCK cells was associated with an increase in their junctional E‐cadherin localization, which is suggestive of stretch‐induced reinforcement of cell barrier integrity. These findings highlight the potential and versatility of StretchView, which can be integrated into various microscopes and serve as a culturing platform for different cell types, facilitating new understanding in the field of mechanobiology and beyond.

## 2 Results and Discussion

### 2.1 StretchView Design and Function

**Figure 1:**
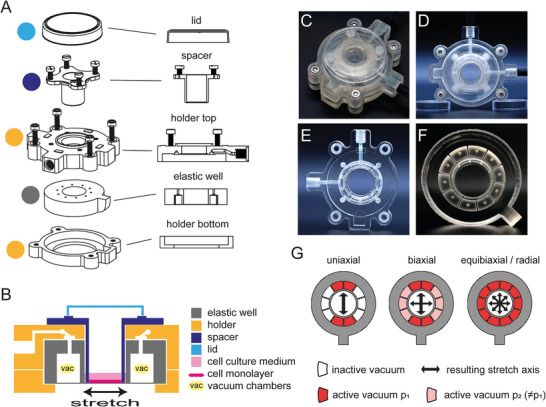
Design of the cell‐stretching system StretchView. A) Technical drawing of the explosion view of the assembly (left) and cut views of the components (right). The PDMS well unit with integrated vacuum actuation chambers features a thin PDMS membrane. The well unit, clamped inside a 3D printed holder, has an open cell culture area with a diameter of 16 mm where the cell culture medium can be added using a pipette. The holder top has an internal channel system that distributes the vacuum to the individual vacuum actuation chambers. On top of the holder, a spacer component for maintaining the focal stability of the membrane during stretch is mounted. The top of the holder is covered by a lid, which is sitting on four vent structures. The lid protects the cell culture from contamination and limits media evaporation. B) Schematical cross‐section of the cell‐stretching system assembly with color‐coded components (also referring to panel A). C,D) Photos of the assembled device from (C) top view and (D) bottom side view. E) Photo of the top holder with an integrated vacuum channel system and two vacuum ports for compatibility with uniaxial and radial actuation. F) Photo of the PDMS device body that comprises the cell culture well, the cell culture membrane, and ten actuation chambers. G) Schematic of device cross section with exemplary stretch modes that can be achieved by activating different combinations of actuation chambers. For uniaxial stretching, opposite chamber pairs along one axis are activated. For general biaxial stretching, two independent chamber sets along two axes can be activated by independent vacuum ports with different pressure magnitudes (p_1_ and p_2_). For equibiaxial/radial stretching (i.e., equal stretching in all axes), all chambers are actuated with the same vacuum. In studies herein, we used uniaxial and radial/equibiaxial stretch modes.

StretchView is a pneumatic cell‐stretching device made entirely out of polydimethylsiloxane (PDMS) and consists of a circular device body with a cell culture well encircled by a ring of comparted vacuum chambers that serve as independent pneumatic actuation elements (**Figure** **1**). The device body is bonded to a thin (100 µm) elastic and highly transparent PDMS membrane, onto which the cells are cultured. The actuation chambers contract when a vacuum is applied to them, pulling on the adjacent cell culture chamber wall and stretching the cell culture membrane bonded to the wall. As the cultured cell monolayer adheres to the underlying matrix, the stretch of the membrane is transferred to the cells (contrary to micropipette aspiration, where cells are stretched directly). The extent of stretch and subsequent strain exerted onto the cells can be adjusted by regulating the strength of the vacuum applied to the actuation chambers. By employing a set of independently controlled actuation chambers (in this case 10 actuation chambers), it is possible to switch between stretch directions during live‐cell imaging without having to readjust the experimental setup (e.g., by reassembling components). For independent control of strains in the x and y direction, a minimum of four chambers (one pair for each direction) is required. Additional chambers allow for intuitive change of axis orientation by selective chamber pair activation without the need for additional control of pressure magnitude. A 3D‐printed holder is used to connect the PDMS device to external hardware (Figure 1A–D). A technical drawing of the explosion view of the assembly with cut views of the components, and a schematical cross‐section are shown in Figure 1A,B. The holder top features a vacuum channel system that connects the actuation chambers to vacuum ports (Figure 1E). The number of vacuum ports and their vacuum chamber assignment can be varied by different holder top designs. The number of vacuum chambers that can be actuated (i.e., are connected to the vacuum) determines the number of possible stretch patterns (each pair of opposed chambers resembles a stretch axis). The design that is used herein has two vacuum ports, allowing for stretching the membrane along one axis (uniaxial stretch), non‐equally along two perpendicular axes (biaxial stretch), and equally over the whole circumference of the cell culture (equibiaxial or radial stretch) as shown in Figure 1G. The holder bottom has a viewing window to ensure access for inverted microscopy with low working distances to the membrane. The central cell culture area can be opened at the top to allow pipette access for membrane treatment and for seeding, manipulation, and infection of cells. This also provides compatibility with end‐point analysis such as western blotting, RNA extraction for RNA‐seq or RT‐PCR, and (immuno)staining followed by quantitative imaging (demonstrated in Section 2.5). A cell‐culture dish lid is used to close the cell culture well in order to prevent media evaporation and lower the risk of contamination, while a vent structure allows for gas exchange. The vacuum pressures in the actuation chambers are controlled by proportional pressure control valves connected to a data acquisition device and a computer with Matlab or Labview (Figure S1, Supporting Information). This setup enables for application of arbitrary waveforms for the applied strain magnitude (e. g. rectangular, trapezoidal, sinusoidal, or previously measured physiological signals). A demonstration of device actuation for uniaxial and radial stretch with rectangular and sinusoidal waveforms is given in Movie S1.

### 2.2 Addressing Defocusing upon Membrane Stretching

A commonly reported problem in live‐cell imaging with pneumatic cell‐stretching devices is membrane movement due to stretch‐induced out‐of‐plane displacement of the membrane, further referred to as z‐displacement. Kreutzer et al and Peussa et al discussed and quantified z‐displacement and introduced a hinge structure as a measure against z‐displacement to reduce it to the order of 45 ± 22 µm compared to initial values of 315 ± 22.5 µm.^[^ ^22^, ^27^ ^]^ However, the angular movement of the hinge introduced a geometrically non‐linear strain‐dependence of the z‐displacement which can be problematic to handle. Despite being a crucial problem hindering live‐cell imaging of stretching cells, z‐displacement in pneumatic devices is rarely discussed in scientific literature. The following sections describe the underlying mechanisms of membrane deflection upon stretch and how StretchView addresses them for long‐term quantitative videomicroscopy of living cells.

#### 2.2.1 A Closer Look at Membrane Deflection Mechanisms

**Figure 2:**
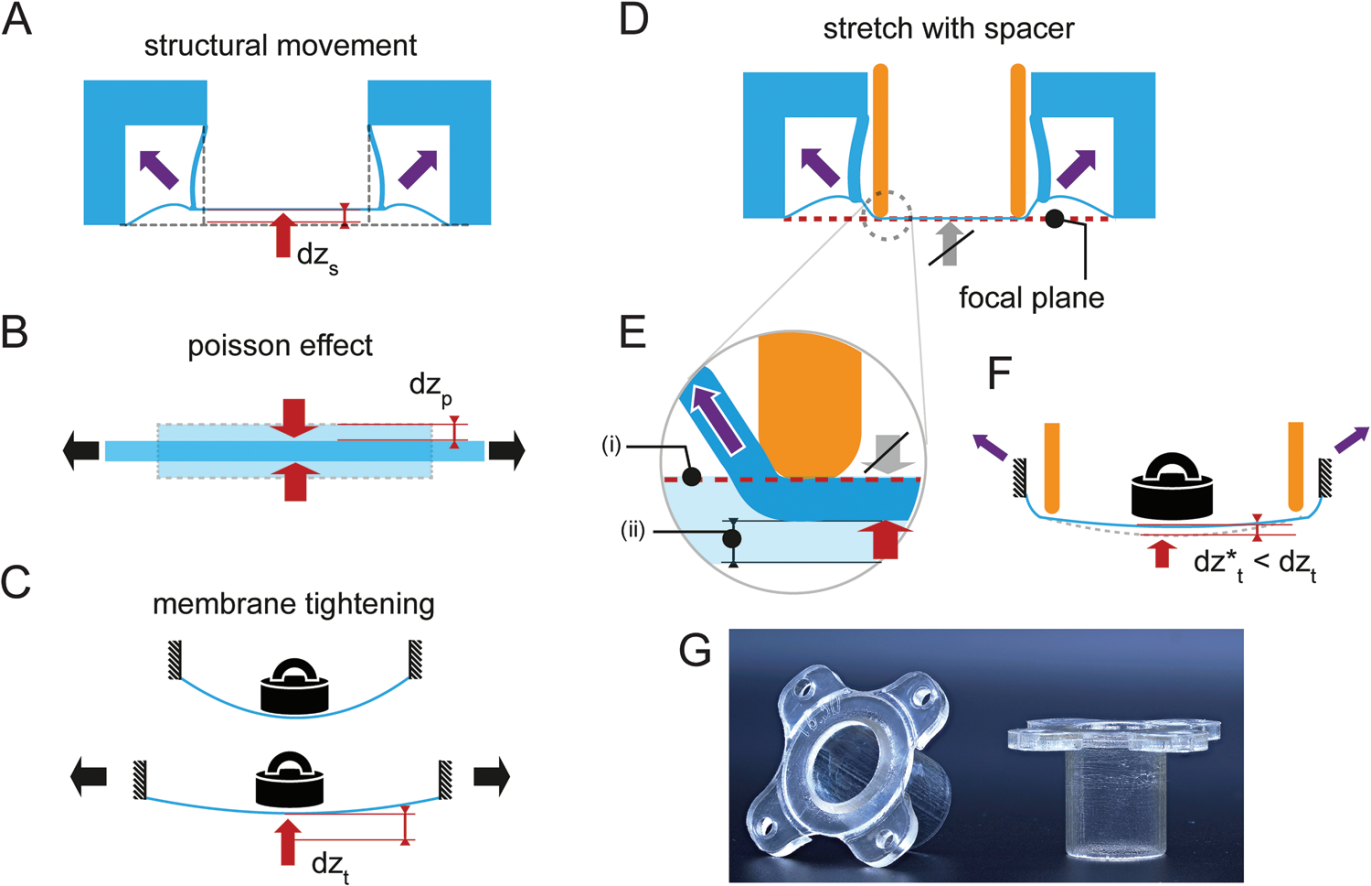
Spacer is used to address out‐of‐plane membrane motion upon stretching that causes defocusing and hinders live‐cell imaging. A–C) Mechanisms that cause out‐of‐plane membrane motion (indicated by red arrow). A) Structural out‐of‐plane motion resulting from the design and arrangement of the actuation chambers resulting in z‐displacement dz_s_. Diagonal forces pulling the membrane up upon stretching are indicated with purple arrows. B) Membrane contraction/thinning caused by the Poisson effect upon membrane stretching. The lateral contraction causes movement of the membrane surfaces, dz_P_ upon stretch. The horizontal displacement component is indicated by black arrows. The top surface of the membrane moves downward if the membrane movement is not restricted. C) Tautening caused upon stretch elevates the membrane (dz_t_) that commonly sags under self‐ and cell‐culture weight (represented by weight symbol). As above, horizontal displacement causing stretching is indicated by black arrows. D–F) The spacer addresses the above‐mentioned types of membrane motion: D) Upon stretch, the membrane slides against the spacer, preventing upward structural motion of the membrane (indicated by crossed out grey arrow). E) The introduction of the spacer also establishes a small amount of membrane pre‐tensioning that corrects z‐displacement caused by the Poisson effect. Pre‐tension is applied to prevent the movement of the top surface of the membrane i) and to direct the Poisson effect‐related thinning motion toward the back surface ii) of the membrane that is not in contact with the spacer. F) Pre‐tension also reduces initial membrane sagging and thus reduces the tautening lift motion to dz^*^_t_. G) Photo of two spacer parts.

Three types of stretch‐induced z‐displacements can be theoretically identified: structural motion upon vacuum chamber actuation that pulls the membrane upward (**Figure** **2**A), Poisson effect motion that leads to perpendicular contraction of the elastomeric membrane upon stretch (Figure 2B), and tautening, resulting in an upward membrane motion (Figure 2C). The most obvious contribution comes from the shape and arrangement of the actuation chambers (Figure 2A). In the StretchView device, the corners of the actuation chambers pull diagonally up upon application of vacuum, elevating the membrane. This structural displacement is indicated in Figure 2A as dz_s_. Besides using a spacer, this deflection mechanism can also be reduced by implementation of hinge geometries in the actuation chamber,^[^ ^27^ ^]^ or eliminated by additional pneumatic chamber volume on the bottom side as realized in the Emulate chip design to add compensational forces by symmetry.^[^ ^17^, ^39^ ^]^ However, the latter approach obstructs the access for inverse objectives with low working distances required for high‐resolution imaging. As previously discussed in the section above, hinge structures can come with the disadvantage of non‐linear z‐displacement characteristics.^[^ ^27^ ^]^

The Poisson effect leads to thinning of the elastomeric membrane that happens perpendicular to the direction of stretch (Figure 2B). This occurs because the elastomeric material's volume is approximately conserved during the deformation. The membrane thinning that takes place during stretching therefore lowers the upper membrane surface, onto which the cell resides and is set as the focal plane, leading to out‐of‐plane images. The Poisson‐induced movement of the top surface (indicated as dz_p_) scales linearly with longitudinal strains and membrane thickness. For example, for a membrane thickness of h = 100 µm, equibiaxial strain of ε = 10%, and Poisson ratio (ν) of 0.5 for elastomers, the deflection of the top surface equals 2.5 µm (following the equation *dz_P_*  =  1/2 νε*h*). Thus, Poisson displacement can also significantly contribute to z‐displacement. Finally, the degree of membrane sagging due to cell culture medium and membrane weight changes upon stretch (Figure 2C). Stretching tautens (tightens) the membrane and results in an upward membrane motion. This effect can be reduced by using a thicker (and stiffer) membrane, a smaller membrane diameter, less media volume, or application of pre‐stretch. The use of stiffer cell culture membranes can be tricky, as the stiffness of PDMS changes over time due to several factors including mechanical stress.^[^ ^43^ ^]^ Finally, using reduced cell culture media volumes or a smaller cell culture well diameter is not always realistic as such changes would have further experimental implications.

#### 2.2.2 The Spacer as an Effective Measure Against Out‐Of‐Plane Membrane Motion

In the StretchView design we considered the use of a spacer as the most promising approach to address all three aforementioned sources of z‐displacement (Figure 2G). The spacer is a distance sleeve that is inserted into the cell culture well to pre‐tension the membrane and limit z‐displacement, similar to the concept of indenters and loading posts used in other systems.^[^ ^19^, ^42^ ^]^ The working principle of the spacer is shown in Figure 2D–F. By lubricating the contact with the periphery of the cell culture membrane, the spacer acts as a slide bearing when the membrane is stretched. This way, it eliminates the structural z‐displacement (Figure 2D), redirects the Poisson z‐displacement to the back surface of the membrane (Figure 2E), and reduces tautening displacement via less sagging due to initial pre‐stretch (Figure 2F).

In experiments performed without using the spacer, we determined z‐displacement values in the order of 300 µm for 5% radial strain, which was measured by manually readjusting the microscope stage until the image was in focus when the membrane remained stretched. Using the spacer, we effectively minimized z‐displacement upon actuation, which enabled us to obtain focused images in both the stretched and unstretched configurations without the need for manual adjustment of the microscope stage. Small displacements (in the order of 10–30 µm) were occasionally observed, likely due to remaining sagging/tightening effects and small variations in spacer height inherent to the production process used (see Section 4). To compensate for these residual z‐displacements, automated imaging was performed in two focal planes (for relaxed and stretched states) that were determined before the start of the live‐cell imaging experiment. In most cases, the focus quality of captured images was not significantly impacted, since we used epifluorescence imaging and not very high magnification objectives (10x, 20x, or 40x with air). Although, such automated stage movement could also be used to somewhat compensate for the high z‐displacements observed upon stretching when the spacer is not used, the range of search for the focal plane would be drastically higher, making it impracticable for image acquisition at short time intervals. Furthermore, it would be expected that when the z‐displacement range is higher, even the perfect focus system of the microscope would fail to identify a focal plane at every membrane deflection.

The use of a spacer that comes in direct contact with the cell culture membrane does not come without limitations. For example, the friction between the spacer and membrane, especially during cyclic stretching, could damage cells residing in the vicinity of the contact area and could lead to cellular monolayer detachment, although we never encountered this problem during our experiments. To completely eliminate the risk of monolayer detachment and cell damage at the periphery, the cell culture membrane can be patterned with cell attachment proteins, in order to define a cell culture area that avoids the contact areas. Another reason to keep cells away from the spacer/membrane contact area is the lubricant itself. In this work, the contact was lubricated with silicone‐free laboratory grease (Borer; Glisseal N). To assess any potential adverse effects of Glisseal N on cell behavior, we applied Glisseal N to the edge of glass‐bottom cell culture wells, as used in the StretchView device, and seeded MDCK cells expressing fluorescent E‐cadherin. We conducted live‐cell microscopy imaging from 24 h to 45 h after cell seeding in the presence of a cell death marker (propidium iodide). After imaging, the cells were fixed and immunostained for the cell proliferation marker Ki‐67. Our results indicate that Glisseal N had no impact on migration, cell death, proliferation, or the intercellular localization of cell‐cell junction protein E‐cadherin (Figure S2, panels A—H, Supporting Information).

### 2.3 Evaluation of Strain Fields

**Figure 3:**
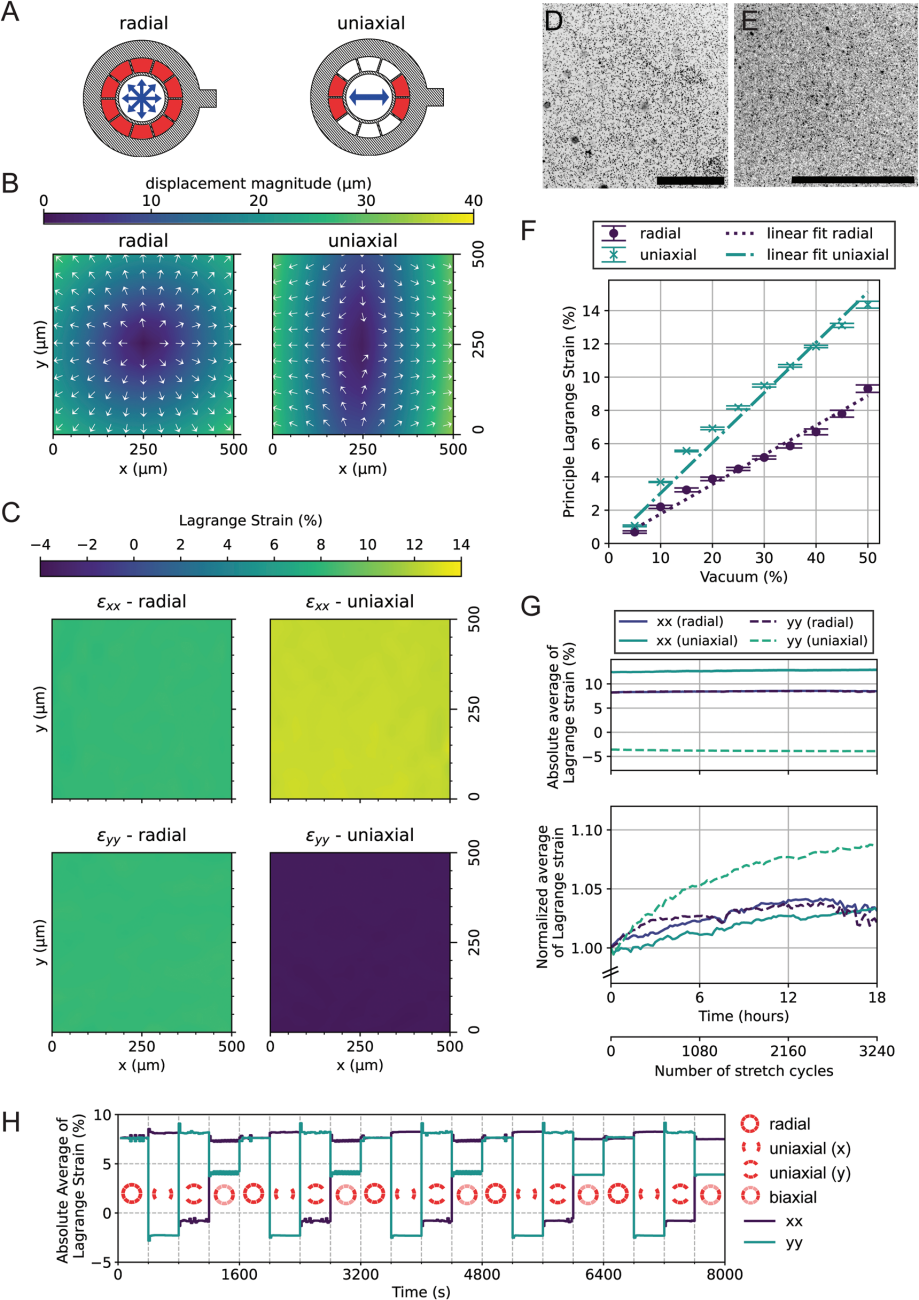
Strain evaluation using Digital Image Correlation (DIC). A) Schematics of actuation patterns for radial and uniaxial stretching (active vacuum chambers in red). Displacement fields and Lagrange strain shown in (B) and (C) are evaluated for radial stretch (left column) and uniaxial stretch (right column). B) Displacement fields in the center of radial and uniaxial stretch. Displacement magnitudes are denoted in the color scale and displacement directions are indicated by white vectors. The arrow lengths are normalized. C) Lagrange strain fields in the principal directions xx (top row) and yy (bottom row) are calculated from the displacement field gradients. For both stretch modes, homogeneous strain fields were obtained in the imaged region. Note that the Lagrange strain in yy for uniaxial stretch is negative due to lateral contraction. D,E) Exemplary images of fluorescent tracer particles were taken with a 10x objective (0.5 µm diameter, D) and a 20x objective (0.2 µm diameter, E). Scale bars: 400 µm. Images of the membrane in (D) were used for the DIC results shown in B and C. F) Strain‐vacuum‐characteristics in radial and uniaxial stretch modes. Vacuum was applied in the range from 5 to 50% and the Lagrange strain was determined via DIC. A linear relationship was observed between the applied vacuum percentage and the resulting strain. Independent measurements were carried out 3 times per stretch mode, using the same device. Symbols represent mean values, error bars represent the standard deviation, and dashed lines represent linear regression curves. G) Exemplary long‐term measurement of temporal strain stability during cyclic radial (8% strain) and uniaxial (12% strain) stretching over 18 h (3240 stretch cycles) with a 20 s period. The upper plot depicts absolute values for average Lagrange strain (spatially averaged over a region of 500 × 500 µm) over time, which show only minor strain fluctuation. The lower plot depicts normalized average Lagrange strain values over time (normalization baseline is the strain value from t = 0). Normalized fluctuations do not exceed 10% with respect to the baseline over the entire time span of 18 h, suggesting the stability of the platform. H) Demonstration of switching between four different cyclic stretch configurations within one experiment. Lagrange strains are spatially averaged (region of interest of 400 × 400 µm) and plotted over time. As indicated by the red schematics (deeper red representing active vacuum chambers), the stretch configuration was altered between the following modes: radial stretch, uniaxial stretch across the x‐axis, uniaxial stretch across the y‐axis and biaxial (strain in x‐axis higher than stretch in y‐axis) stretch. Each mode was maintained for 400 s (20 stretch cycles with a period of 20 s) and the whole pattern was repeated five times.

In contrast to motorized cell‐stretching systems, where the stretch is controlled by directly pulling the membrane horizontally at known increments, the use of pneumatic systems creates the need for pressure‐strain calibration, i.e., for every experiment it is necessary to establish the amount of applied vacuum that corresponds to specific strains. By acquiring focused images in both the relaxed and stretched states of the membrane, we were able to measure in‐plane membrane deformation and strain by imaging speckle patterns on the membrane and performing Digital Image Correlation (DIC). We imaged the deformation of these speckle patterns upon uniaxial and radial stretch (**Figure** **3**A), focusing on the region around the stretching center (Figure 3A–C). Microscale speckle patterns were previously applied by covalently attaching fluorescent tracer particles to the PDMS membrane surface (Figure 3D,E). As expected, the deformation is zero at the center of stretching, as can be seen in the exemplary displacement plots in Figure 3B. For both radial and uniaxial stretch, the Lagrangian strain in the principal directions xx and yy was homogeneous over the whole region of interest (Figure 3C). In the exemplary measurement in Figure 3C, the radial stretch at 0.65 bar or 35% vacuum resulted in a strain field with a magnitude of 8% in xx‐ and yy‐direction. Uniaxial stretch at 0.65 bar or 35% vacuum resulted in a uniaxial strain field with 12% Lagrangian strain in xx‐direction and −4% Lagrangian strain in the yy‐direction. The negative strain in the yy‐direction is caused due to lateral contraction.

#### 2.3.1 Strain‐Vacuum Characteristics

Using the analysis method described above, curves for strain‐vacuum characteristics were measured for vacuum pressures ranging from 0.95 to 0.5 bar (or respectively 5–50% vacuum). The measurements were carried out on one device with N = 3 repetitions. Similar to other published devices,^[^ ^22^, ^27^ ^]^ we observed a nearly linear relationship between strain and vacuum for both uniaxial and radial stretch (Figure 3F). At a vacuum of 50% we observed 14% and 9% Lagrange strain for uniaxial and radial stretch respectively.

#### 2.3.2 Strain Stability During Long‐Term Stretching Experiments

Above, we used DIC to track fluorescent particles attached to the stretching PDMS membrane in order to calculate the strain induced on the cell culture membrane during pneumatic actuation. Most published examples of pneumatic cell‐stretching devices use similar approaches to characterize the pressure‐strain relationship prior to experiments,^[^ ^27^, ^36^ ^]^ but do not acquire strain data during the experiments. Continuous strain measurement is particularly important during very long experiments, as elastomeric membranes under cyclic load are subject to softening mechanisms. Although the stiffness of an elastomer stabilizes within the first couple of cycles (a mechanism referred to as the Mullins effect),^[^ ^44^ ^]^ the stiffness of PDMS can be significantly reduced in the range of several hundred to thousands of cycles.^[^ ^45^ ^]^ As time scales of cellular processes like extracellular matrix remodeling can range from hours to days, imaging over many stretch cycles under stable stretch conditions is a relevant requirement for live‐cell imaging on stretching in vitro cell culture systems. With such experiments in mind, we evaluated the strain stability of StretchView. For that, we performed cyclic membrane stretching for 18 h (3240 stretch cycles with a period time of 20 s and rectangular waveform) and applied DIC on images of fluorescent tracer particles taken in 10‐min intervals at the stretched and relaxed membrane center, in order to evaluate the temporal stability of the resulting strain field. We computed Lagrangian strain fields in xx‐ and yy‐direction and then spatially averaged them over a region of interest of 500 × 500 µm size for each imaging interval. The resulting averaged strains are given as absolute and normalized values in Figure 3G for radial and uniaxial stretching. The average Lagrange strains remained stable over the course of the experiment, whereby the strain values at the first stretch cycle are 8.2% strain in xx‐ and yy‐direction for the radial and 12.5% in xx‐direction and −3.6% in yy‐direction for the uniaxial configuration. As can be seen from the normalized average strain plots, the maximum relative changes of the Lagrange strains with respect to the first stretch cycle (normalized to 1) stay below 10% for the investigated time span (for radial: 4.2 and 3.6% relative fluctuation for strain in xx‐direction; for uniaxial: 3.6 and 8.8% relative fluctuation for strain in xx‐ and yy‐direction). This corresponds to the maximum absolute changes of measured Lagrangian strains by 0.4%. These results demonstrate that StretchView is capable of maintaining stable strains during long‐term live‐cell imaging experiments.

#### 2.3.3 Switching between Radial, Uniaxial, and Biaxial Stretch

Switching between stretch directions (equibiaxial/radial, uniaxial, and biaxial) can be a relevant parameter for the study of fundamental biological processes, but has received little attention so far. The multiple vacuum ports in StretchView allow for the application of different axial stretch configurations within one single experiment. In order to demonstrate this functionality, we applied a pattern where we alter between four stretch configurations: radial (or equibiaxial), uniaxial along the x‐direction, uniaxial along the y‐direction, and biaxial with higher strain in the x‐direction. Each cyclic stretch configuration is maintained for five periods. As described in the previous section, we applied DIC on period‐resolved image pairs (relaxed and stretched) and analyzed spatial averages of Lagrange strains over time. The resulting average Lagrange strains for each applied stretch configuration can be seen in Figure 3H. For the radial configuration, the strain value is 8% in both x‐ and y‐direction. In both uniaxial configurations, the quantified axial strain is 8%, whereby the compression strain in the lateral direction is −2 and −1% for the uniaxial stretch mode in x and y, respectively. We hypothesize that this difference in lateral contractions originates from the 10‐chamber design or from varying friction between the spacer and the membrane. For the biaxial configuration, the applied strains are 8 and 4% for the x‐ and y‐direction, respectively. We repeated this alteration pattern three times and observed reproducible strain values.

#### 2.3.4 Evaluation of Strain Fields During Live‐Cell Videomicroscopy

**Figure 4:**
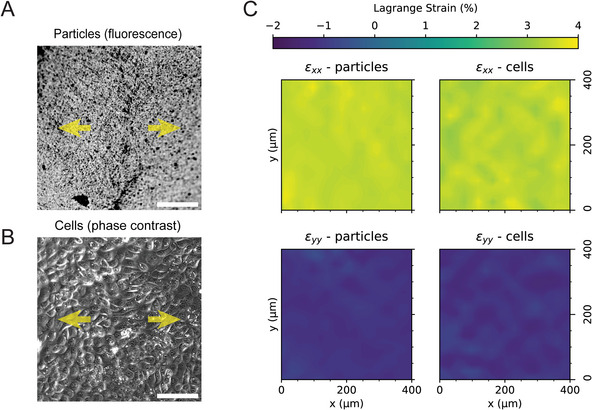
Comparison between strains that were calculated using imaged tracer particles and strains that were calculated using imaged cells. A) Fluorescent particles bonded to the PDMS membrane and (B) MDCK cells seeded on the same collagen I‐coated membrane. The membrane is stretched uniaxially (indicated by yellow arrows). Cells were visualized using phase contrast and tracer particles in fluorescence. Scale bars: 100 µm. C) Lagrangian strains derived by DIC on images of fluorescent tracer particles or phase contrast are in good agreement, suggesting that phase contrast images of cells can be used for strain calculation.

Simultaneous cell imaging and membrane strain measurement is possible, and can be simply achieved by imaging the tracer particles attached to the membrane and the cells in separate channels. However, given that cells are themselves attached (i.e., adherent) to the stretching membrane, we can bypass covalent tracer particle attachment by directly analyzing acquired time‐lapse phase contrast images of cells for strain calculation via DIC. A key requirement for this is that well‐focused images are acquired when cells are in the relaxed and stretched states, which was demonstrated to be possible using StretchView. As a first step toward evaluating whether particle‐calculated strain corresponds to cell‐calculated strain, we cultured MDCK cells in monolayer on PDMS stretching membranes onto which fluorescent tracer microparticles were first covalently attached. After imaging the particles and cells in separate channels, we performed DIC and compared the Lagrange strain results from the imaged particles to Lagrange strains calculated from phase contrast images of cell displacements. As shown in **Figure** **4**, there was good correspondence between particle‐calculated strain and cell‐calculated strain. Therefore, in order to simplify the experimental process, cell displacements were used to evaluate strain in the experiments discussed in the following sections of this article.

### 2.4 Live‐Cell Imaging of Epithelial Cells in Monolayer during Cyclic Stretching

Once the functionality and long‐term stability of StretchView were established, we used it to stretch and image MDCK cells in monolayer to demonstrate that long‐term multichannel microscopy is feasible with cells residing on the stretched PDMS membrane, as shown in Movie S2, where MDCK cells expressing E‐cadherin‐RFP were radially stretched for 12 h at 5% strain and a 20 s period. During this time, we captured phase‐contrast and E‐cadherin fluorescence images every 50 s, both in unstretched and subsequently stretched configurations. This illustrates the compatibility of our StretchView device with extended live‐cell microscopy. In the following sections, we present initial investigations on how cyclic cell‐stretching impacts cell kinematics, E‐cadherin localization, and cell shape parameters based on phase‐contrast and E‐cadherin fluorescence images.

#### 2.4.1 Characterization of Cell Kinematics during Cyclic Stretching

**Figure 5:**
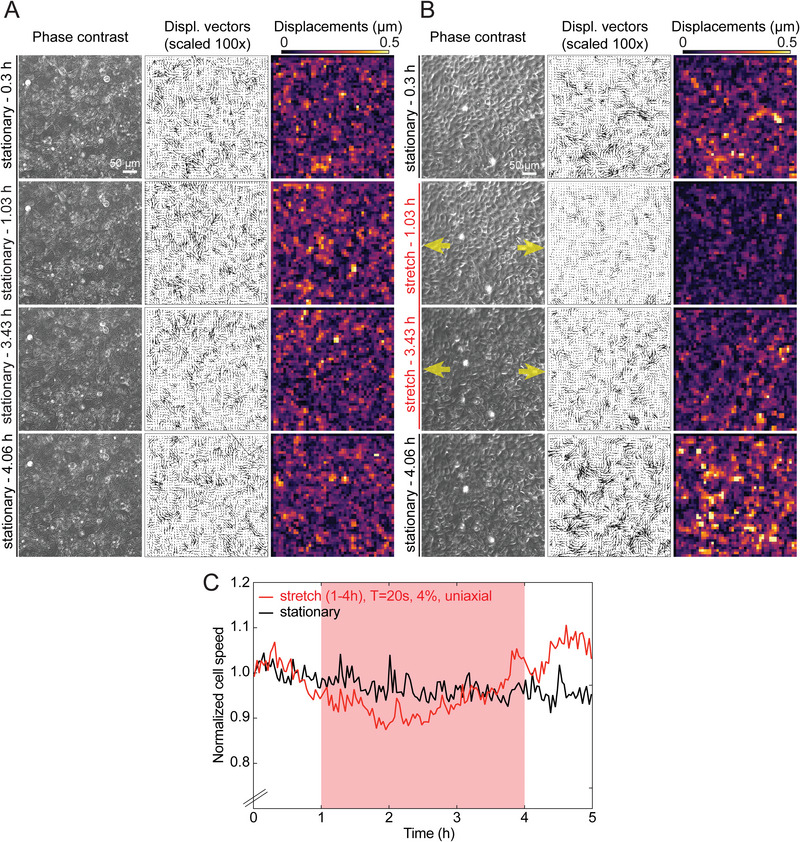
Epithelial MDCK cells slow down their motility when they are subject to cyclic uniaxial stretching. A) Representative time‐lapse microscopy images of MDCK cells in monolayer under stationary conditions or (B) when subject to 4% uniaxial strain with T = 20 s along the direction indicated by the yellow arrows. Columns show: phase contrast image; cell displacement vectors (black arrows) scaled 100x; and magnitude of cell displacements (in µm). Rows show four different time points along the time‐lapse acquisition, which in the case of stretched cells (B) coincide with a timepoint before stretching (stationary–0.3 h, 1st row), a time point after stretching is initiated (stretching −1.03 h, 2nd row), another one ≈2.5 h later (stretching–3.43 h, 3rd row), and a time point that refers to the first frame after stretching has been stopped (stationary −4.06 h, 4th row). Note that subsequent phase contrast images of cells in the “unstretched” configuration were compared and the frame interval used to perform particle image velocimetry (PIV) is 2 min irrespective of whether cells were subject to stretch or not. C) Plot of mean cell speed normalized to the first timepoint versus time (h) for representative examples of cells under stationary conditions shown in panel A (black line) and under stretched conditions shown in panel B (red line). Note that in the first and last hour of the 5 h long acquisition, cells were under stationary conditions and were only subjected to 4% uniaxial strain with T = 20 s from t = 1–4 h (see red shaded area). Absolute cell speed values (µm h^−1^) versus time (h) without normalization are shown in Figure S3 (Supporting Information).

Epithelial cells forming a confluent monolayer are typically quiescent (i.e., cell motility is reduced) which is essential for the maintenance of their barrier integrity. However, insults ranging from compressive forces (e.g., during bronchospasm of airway epithelial cells) to bacterial infection, can render them motile, which is often associated with reduced barrier integrity.^[^ ^46^, ^47^ ^]^ To assess whether and how stretching impacts cell motility, we acquired time‐lapse phase contrast microscopy images of MDCK cells in monolayer for 1 h prior to stretching, followed by cyclic stretching for 3 h at 4% strain and a rectangular waveform with a period time of 20 s. The time interval between stretching cycles was 30 s. We chose these specific parameters of strain magnitude and period because they are close to the physiological values reported in vivo for intestinal epithelial cells.^[^ ^39^ ^]^ We opted for MDCK cells since they form polarized and homogenous monolayers in culture and have been vastly used as a model to predict drug transport from the intestinal lumen into the bloodstream.^[^ ^48^ ^]^ To avoid many imaged cells moving out of the field of view (FOV) upon stretching, we aligned the center of the FOV with the center of stretching and tracked cells residing away from the FOV boundaries. As in Section 2.3, to quantify cell kinematics and calculate mean cell speed we used an image correlation technique similar to particle image velocimetry (PIV) on the phase contrast images as done previously.^[^ ^49^, ^50^ ^]^ Interrogation windows of 32×16 px (window size x overlap) yielded a resolution of ≈8.8 µm in the resulting displacement field. **Figure** **5**A,B shows the phase contrast images used for PIV analysis. It is worth pointing out that although the images in panel A show less distinct cell boundaries compared to the images in panel B, the PIV analysis remains robust by calculating displacement fields through correlated, overlapping windows without the need for cell boundary detection. Images taken at the unstretched state were used as controls when characterizing cell displacements and average cell speed overtime, including during stretching.^[^ ^49^ ^]^ We found that control cells never exposed to stretch, were in a steady state, and exhibited minimal fluctuations in their displacements and cell speed over time (Figure 5A,C; Figure S3A, Supporting Information). However, cells exposed to cyclic stretch, gradually showed a decrease in their speed during stretch, reaching a minimum at ≈1 h post‐stretch initiation of ≈10% lower speed compared to the beginning of the recording or compared to cells under stationary conditions (Figure 5B,C; Figure S3B, Supporting Information). However, cells gradually recovered their speed within the course of the 3rd h of stretch. Moreover, after cessation of stretch we continued acquiring images to assess whether cells would keep any sort of mechanical memory of the past stretch exposure or would alternatively return to the same speed as before stretching. Interestingly, we found that upon stretch cessation cell speed increased as compared to before or during stretch application. To unequivocally confirm the effects of stretch on the kinematics of cells in a monolayer, more systematic investigations should be performed. However, these preliminary proof‐of‐concept experiments suggest that, first, using our stretching device, we can accurately characterize cell kinematics over long times of cyclic stretch, and second, there are likely interesting alterations in cell movement resulting from cyclic stretch, which we can analyze using the described easy‐to‐implement approach. Moreover, it is worth noting that these results are consistent with what Hart et al. reported previously using a pneumatically‐actuated PDMS‐based cell‐stretching device.^[^ ^26^ ^]^ In this work MDCK cells in monolayer were stretched uniaxially at 15% strain and were observed for 7 h via time‐lapse microscopy. Using their stretching device, the optical focus had to be adjusted manually during stretching and a low magnification objective was used (5x). Although the strain applied was higher than the one used herein, it was found that cells during the first 2 h of stretch reduced by 20% their average speed as compared to cells exposed to no strain but then eventually returned to levels similar to those before application of stretch. This is also consistent with the notion that stretching cells in monolayer can increase their quiescence possibly due to reinforcement of intercellular adhesions that restrain their motion. However, additional systematic investigations are needed to more definitely examine how varying stretching strain, direction, and frequency impact kinematics of cells in monolayer and whether additional parameters such as cell density modulate the sensitivity of cells to respond to stretch.

#### 2.4.2 Characterization of E‐Cadherin Localization during Cyclic Stretching

**Figure 6:**
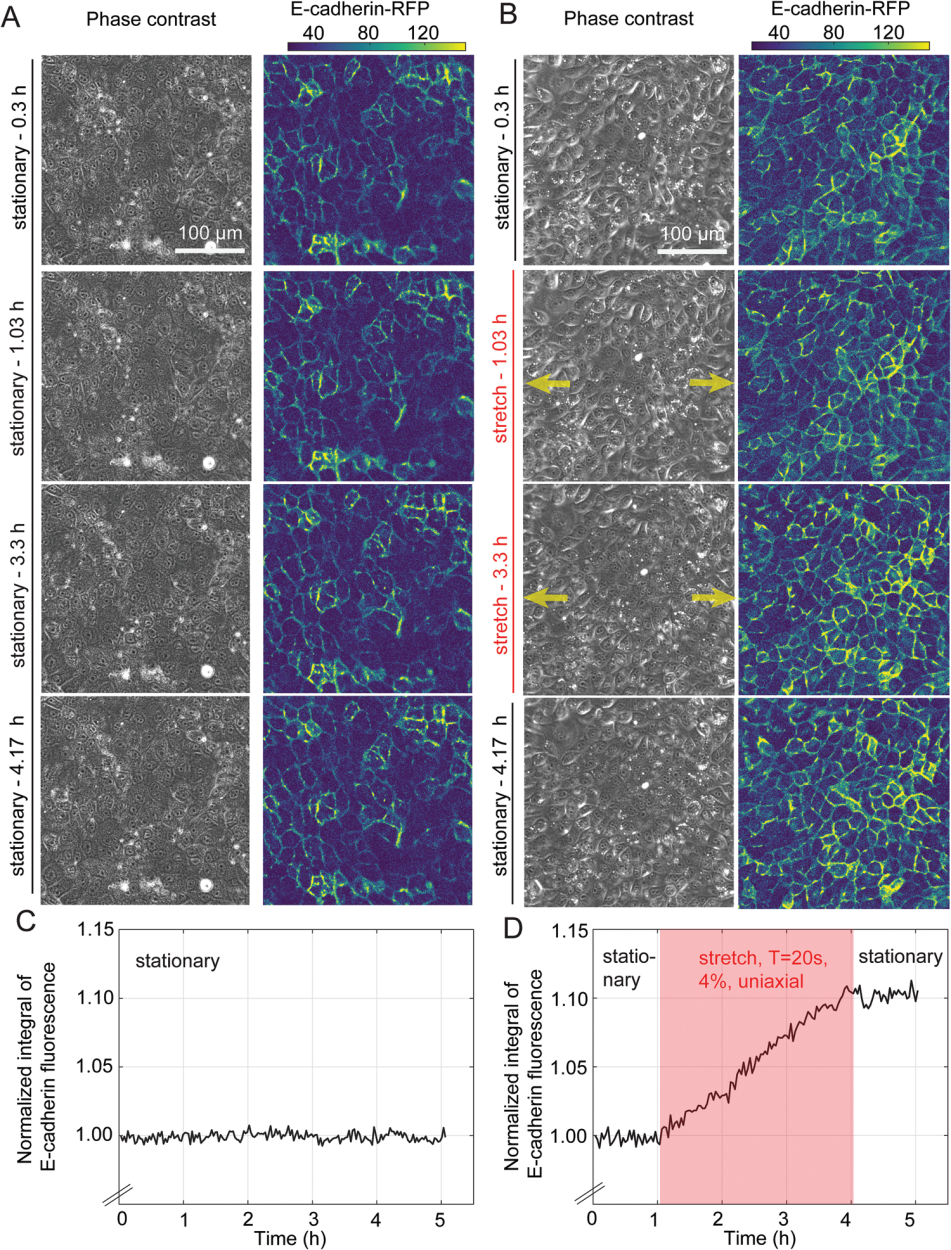
Epithelial MDCK cells show a progressive increase in the localization of E‐cadherin at cell‐cell junctions when subject to periodic uniaxial stretch. A,B) Representative time‐lapse epifluorescence microscopy images of MDCK cells in monolayer constitutively expressing E‐cadherin‐RFP under stationary conditions (A) or when subject to 4% uniaxial stretch with T = 20 s along the direction indicated by the yellow arrows (B). Columns show phase contrast images (1st column) and corresponding background‐corrected E‐cadherin fluorescence intensity images (2nd column). Rows show 4 different time points along the time‐lapse acquisition (t = 0 h is when image acquisition starts), which in the case of stretched cells (B) coincide with a time point before stretching (stationary–0.3 h, 1st row), two time points during stretching, with one immediately after stretching is initiated (stretching – 1.03 h, 2nd row) and another one ≈2h later (stretching–3.3 h, 3rd row), and a last time point that refers to the first frame after stretching was stopped (stationary – 4.17 h, 4th row). C) Plot of normalized integral of E‐cadherin fluorescence intensity versus time for the representative example of cells under stationary conditions shown in panel A. Integration was performed over the whole FOV and normalization was performed with respect to the first image of the acquisition, so that differences in fluorescence intensity over time become apparent. D) The same plot as in panel C but referring to the representative acquisition shown in panel B. Note that in the first and last hour of the 5 h long acquisition cells were under stationary conditions, and were subjected to 4% uniaxial stretch with T = 20 s only from t = 1–4 h (see red shaded area). Similar observations were made for radially stretched cells (see Figure S4, Supporting Information).

Next, we wondered whether this decrease in cell motility during the first 2 h of cyclic stretch could result from alterations in intercellular adhesion organization that could restrain cell motion and whether we could assess that in real‐time using our StretchView device. To that end, we seeded MDCK cells expressing E‐cadherin‐RFP (an important cell‐cell adhesion protein) on the functionalized PDMS membrane of StretchView and let them form a monolayer.^[^ ^51^ ^]^ We then performed multi‐channel live‐cell epifluorescence microscopy for several hours for cells exposed to either cyclic 4% uniaxial or 5% radial stretch or cells under stationary conditions (no stretch). By performing image processing on the time‐lapse images of E‐cadherin fluorescence, we discovered that unlike cells not exposed to stretch whose E‐cadherin intercellular localization appeared to be at steady state (**Figure** **6**A,C and Movie S3), cells stretched either uniaxially or radially showed a quasilinear increase in the pericellular localization of E‐cadherin during the 3–5 h course of cyclic stretch (Figure 6B,D; Figure S4, Movies S4, S5, Supporting Information). To ensure that this linear increase was specifically due to stretching and not influenced by changes in cell density, we first imaged the cells for 1 h under stationary conditions before applying stretch. During this pre‐stretch phase, there were no changes in E‐cadherin localization (Figure 6D). Notably, after ≈1 h of imaging post‐stretch (i.e., after cessation of exposure to cyclic stretch), E‐cadherin fluorescence stabilized at the peak level achieved after 3 h of stretching, with no further increase (Figure 6D). These proof‐of‐concept findings suggest for the first time that cyclic exposure of epithelial cells in monolayer to stretch leads to a monotonic increase in junctional E‐cadherin localization (at least in the 3 h time frame that cells were stretched). This increased E‐cadherin localization at intercellular junctions is associated with decreased cell motility over the first 2 h of stretch. However, it is still to be uncovered what exactly occurs after the 2 h stretch period and why both junctional E‐cadherin localization and motility increases afterward. Interestingly, a recent study showed that E‐cadherin is required for the reduction of migration speed in response to 0.1 Hz cyclic stretching.^[^ ^38^ ^]^ Increased E‐cadherin intercellular localization also most definitely hints at increased barrier integrity of the monolayer, since E‐cadherin is a key component of adherens junctions and organizer of the epithelial barrier.^[^ ^52^, ^53^ ^]^ Consistent with this finding, previous studies on endothelial cells and human induced pluripotent stem cells exposed to low magnitudes (<5% strain) of cyclic stretching are also supportive of amelioration of barrier integrity and/or increased mechanical strength due to increased expression of focal adhesion and/or adherens junction markers.^[^ ^12^, ^54^, ^55^, ^56^ ^]^ Moreover, our results demonstrate that StretchView is compatible with real‐time epifluorescence imaging of cells and thus monitoring and characterization of cytoskeletal dynamics. To our knowledge, completely automated live‐cell imaging during cyclic stretching was never demonstrated before at these timescales.

#### 2.4.3 Characterization of Cell Shape Parameters during Cyclic Stretching

**Figure 7:**
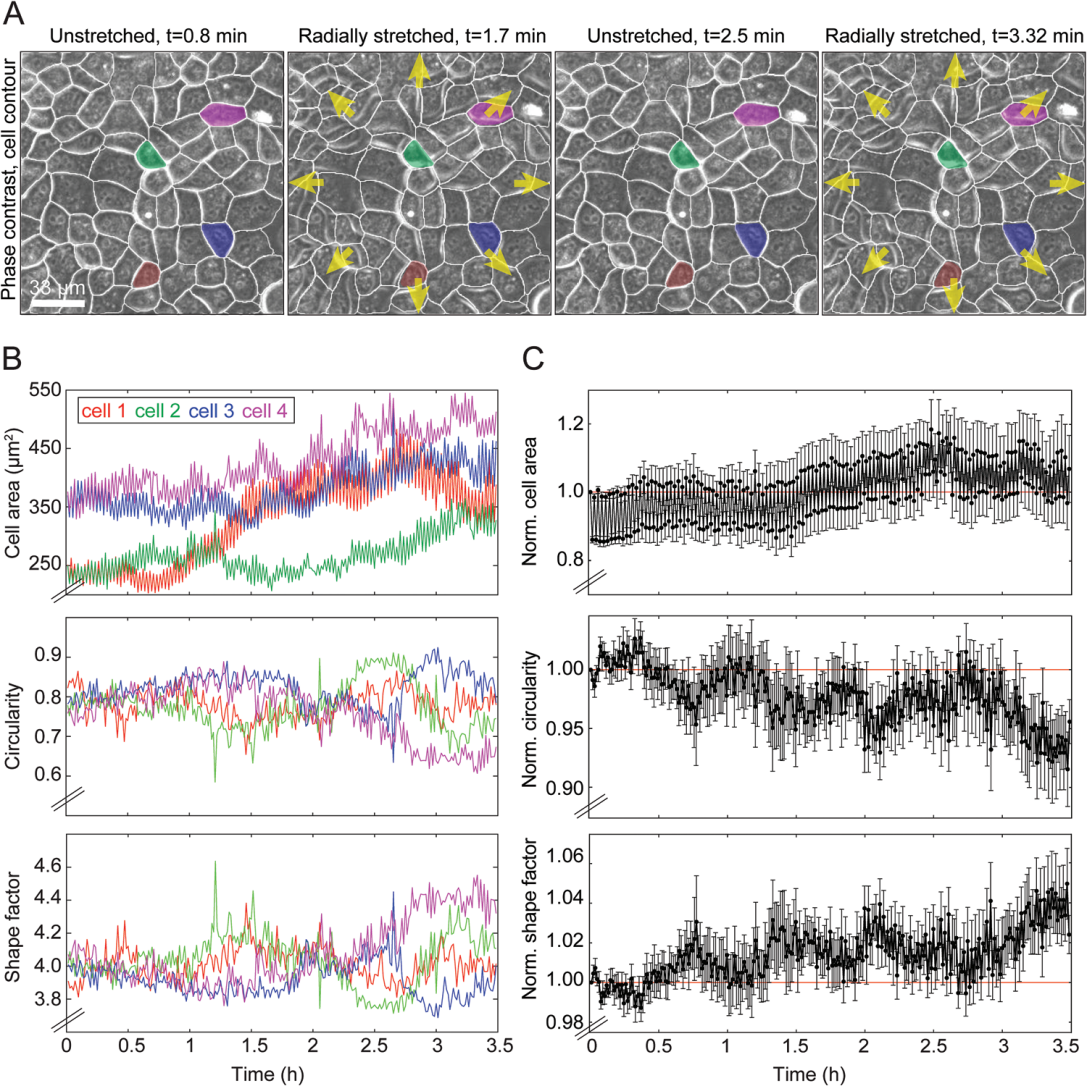
Epithelial MDCK cells subject to cyclic radial stretching show an increase in their area and shape factor but a decrease in circularity over the course of time. A) Representative phase contrast images of MDCK cells in monolayer subject to 5% radial stretch with T = 20 s indicated by yellow arrows. The overlaid white contour indicates the borders identified for the cells at t = 0.8 min (first representative instance of time shown). Four segmented and tracked cells are color‐coded for the time sequence shown and their specific parameters are calculated from the images and are shown in panel B using the corresponding colors. B) The upper plot shows the cell area (µm^2^) versus the time of the four cells colored in panel A. The Middle plot shows the circularity versus time of the same four cells and the bottom plot shows their shape factor versus time. Cell area appears to increase over time as cells are subjected to cyclic stretching. Conversely, cell circularity and shape factor appear to remain relatively unchanged. C) Plot of normalized mean cell area (upper), circularity (middle), and shape factor (bottom) versus time for N = 11 individually tracked cells, the recording of four of which is shown in panel A. Values for each of the three parameters of each cell were normalized relative to the value at t = 0 s (first frame from time‐lapse recording). Vertical bars show the standard error of the mean (SEM). In the normalized plots that contain data from a larger number of cells it can be seen that similar to cell area, cell shape factor also increases after cyclic stretch, while cell circularity decreases.

Changes in cell shape can critically impact cell function and are often predictive of collective changes in tissue behavior during exposure to external cues.^[^ ^57^, ^58^ ^]^ To characterize morphometric changes in epithelial cell shape upon exposure to cyclic stretch, we used the ImageJ plugin Trackmate on the phase contrast image of cells to segment and track individual cells over the course of hours (**Figure** **7**A and Movies S6, S7).^[^ ^59^, ^60^ ^]^ As expected, the cell area oscillated by ≈10% during exposure of cells to cyclic radial stretching, with this number probably depending on the extent to which cells are stretched (Figure 7B,C, top row). We did not observe such oscillations in the circularity of single cells, a parameter that attains a value of 1 for perfectly circular cells and lower values for more elongated cells (Figure 7B, middle row). Likewise, there were no oscillations in the cells’ shape factor (or index), which is the ratio of the cell perimeter to the square root of area, and which can be thought of as a measure of the shear rigidity of cells (Figure 7B, bottom row).^[^ ^51^, ^61^ ^]^ Isotropic solid‐like hexagonal cells have a shape factor close to the critical value of 3.81, while more fluid‐like cells that exhibit reduced rigidity have shape factors of higher value.^[^ ^62^ ^]^ Despite the lack of periodic oscillations directly induced by stretching, when we looked into the average behavior of many cells (N = 11 cells) by normalizing for each cell the respective parameter values at the zero timepoint (unstretched cells), we observed that circularity appeared to decrease over ≈2 h of cyclic stretch applications (i.e., cells get more elongated) as seen in Figure 7C in the middle row. Similarly, the shape factor increased, which suggests a gradual decrease in shear rigidity induced by cyclic stretching (Figure 7C, bottom row). Consistent with cells under stationary conditions being at a steady state in their kinematics, we found no alterations in any of the examined cell shape parameters for those cells, which reinforces that the changes in cell shape observed for cells exposed to stretch are meaningful and not due to random cell fluctuations (see Figure S5A,B, Supporting Information). These findings are consistent with previous end point studies (i.e., not dynamic characterization of cell shape changes) of fibroblasts subjected to cyclic stretch, where cell elongation (aspect ratio) increased significantly 24 h after stretching.^[^ ^63^ ^]^ Similarly, adipose‐derived stem cells exhibited increased cell shape factor after being stretched cyclically.^[^ ^64^ ^]^ Although the findings presented here are initial, they underline the wealth of information that can be extracted using StretchView, and demonstrate the potential application of this device to the examination of how cellular stretching might impact cellular and tissue kinematics, shape morphometrics and dynamics in space and time.

### 2.5 End‐Point Measurements

As stated previously, StretchView's open‐top design ensures compatibility with various end‐point measurement techniques, including western blotting, RNA extraction for RNA‐seq or RT‐PCR, and (immuno)staining followed by quantitative imaging. To demonstrate the feasibility of performing end‐point measurements, we fixed MDCK cells expressing E‐cadherin‐GFP after 22 h of biaxial stretching (8% in x and 2% in y) and used fluorescently‐conjugated phalloidin to visualize F‐actin. The samples were successfully imaged using confocal microscopy, and the resulting orthogonal views can be further inspected in Figure S5C (Supporting Information). Such an approach could be used in future studies for the investigation of the influence of mechanical stretching on different cytoskeletal structures, as well as mechanosensitive transcription factors and protein kinases).^[^ ^65^, ^66^ ^]^

### 2.6 Discussion of Stretch Period, Strain Rate, and Strain Waveform

Control over strain rate or stretch frequency is relevant in cell stretching experiments, as epithelial cells demonstrate strain rate‐dependent responses. Generally, the mechanical behavior of biological cells is classified as viscoelastic, meaning it is influenced by the strain rate.^[^ ^67^ ^]^ Specifically, epithelial cells modulate their tension state over time scales of minutes in a manner that is dependent on strain rate and primarily governed by actomyosin activity.^[^ ^68^, ^69^ ^]^ A recent study performed by Dow et al. indicates that the migratory response of epithelial cells is also sensitive to frequency and strain rate, with lower frequencies (0.1 Hz in sinusoidal stretching) leading to a downregulation of migration.^[^ ^38^ ^]^ The application of high strain rates is of relevance for simulating traumatic injuries to cells in vitro.^[^ ^70^ ^]^ The minimum applicable period time may be experimentally limited by image acquisition requirements, and is physically limited by the inertia of the pressure control system (in the case of pneumatic stretching systems) and the device design. The physical limit to the strain rate (and therefore the maximum frequency or minimum possible period time) is determined by a number of system characteristics, these being the volume to be evacuated for actuation (total volume of vacuum chambers and vacuum lines), the flow resistance of the vacuum lines, the flow rate of the vacuum source, and the responsiveness of the vacuum control valve. Together, these characteristics determine the time difference between the set pressure value and the actual pressure value, the latter of which is related to the strain exerted by the pneumatic chambers. In most studies involving cell stretching, frequencies between 0.01 and 10 Hz are used, covering a range of (patho)physiologically relevant frequencies, corresponding to periods from 100 down to 0.1 s.^[^ ^63^ ^]^ In our experiments, we primarily applied a rectangular waveform and a period time of 20 s (frequency of 0.05 Hz), both to stay within this frequency range and to allow adequate time for multi‐channel image acquisition during each stretch cycle (phase contrast and fluorescence channels). A higher number of channels and/or a longer exposure time increases the time required for microscope and camera operation per cycle, which limits the minimum possible period time that is applicable without pauses for image acquisition. If the experimental study requires high frequencies (i.e., short periods), imaging pauses need to be considered to avoid motion blur during exposure.^[^ ^33^ ^]^ An alternative approach to achieve multichannel imaging for short stretch periods could be a split multichannel acquisition where different channels are measured in different consecutive stretch periods. Based on measurements of the pneumatic response of our system (Figure S6, Supporting Information), a higher frequency (1 Hz) is possible with StretchView, which is discussed in detail below and demonstrated in Movie S1.

#### 2.6.1 Pneumatic Response Characteristics and Estimation of Strain Rate

To characterize the pneumatic response and to estimate the strain rate during the experiments, we measured the pressure at the pressure valve of the pneumatic control setup and correlated it with the DIC‐based strain measurements. A rectangular pressure waveform used here, theoretically results in an instantaneous pressure jump with an infinite slope. In practice, the control signal has to be sampled in time and the responiveness of the valve actuation is finite as well, resulting in a finite slope. In our case, the slope in the input signal was 17.5 bar s^−1^ and the signal was sampled in steps of 10 ms. Figure S6 (Supporting Information) shows the measurement of actual pressure response during radial actuation in the experiment associated with Figure 3H. The required time of the evacuation process from 0 to 35% (our default set vacuum in this study) was ≈300 ms, as shown in Figure S6 (Supporting Information), Panel C. However, with the applied set pressure slope of the rectangular pressure signal, this is followed by an overshoot and a stabilization phase, leading to a total response time of 1.5 s. This stabilization time was taken into account for the timing of automated image acquisition in this work. By differentiation, we obtained the actual temporal pressure slope (pressure change rate). We measured that the jump from 0% to 35% vacuum (pressure 1 to 0.65 bar) took place at an actual pressure change rate of ≈2 bar s^−1^, which correlates to a strain rate of 46% s^−1^ (in the corresponding experiment we measured a strain of 8% by DIC at 35% vacuum). For return to atmospheric pressure, a pressure change rate of 1.3 bar s^−1^ and a correlated strain rate of 30% per second was measured. This discrepancy between the strain rate at the stretch and the relaxation step is a drawback of the rectangular wave signal. Based on these observations, we recommend using a trapezoidal waveform over a rectangular waveform for systematic studies with defined strain rates. This allows for a controlled vacuum ramp that can avoid overshoots in the pressure signal, resulting in a more stable strain rate at both stretch and relaxation phases and a well‐defined strain plateau. Based on the measured evacuation time of ≈300 ms, we anticipate a practically applicable frequency of up to 1 Hz, given the application of waveforms that avoid pressure overshoot response and improve pressure stabilization. If more dynamic actuation is required, a setup with a higher flow rate of the vacuum source (stronger vacuum nozzle or pump) should be considered. In Movie S1, actuation with frequencies of 0.5 and 1 Hz is showcased with a rectangular and sinusoidal waveform.

## 3 Conclusions and Future Perspectives

In this work we introduce StretchView, a cell‐stretching platform that allows, for the first time, automated and long‐term live‐cell imaging of cyclically stretched cells. The device is comprised of an elastic PDMS well with a thin PDMS cell culture membrane, encircled by actuation chambers that allow multiaxial stretching. Experimental conditions such as the stretch direction (equibiaxial/radial, biaxial, uniaxial, or combination), duration of experiment, stretch period, and stretch magnitude can be adjusted, and long imaging experiments (demonstrated up to 18 h) can proceed without further user input. This is a significant improvement over other studies with cell‐stretching devices, which typically required manual image refocusing between stretching cycles, hindering automated image acquisition at regular time intervals. In StretchView, focal stability is maintained by a spacer that minimizes out‐of‐plane membrane motion upon stretching. This allows the acquisition of in‐focus images of the cyclically stretched membrane surface in both the relaxed and stretched configurations, even during experiments with short stretch periods (e.g., 10 s stretch and 10 s release). As stable, long‐term imaging during stretch was our primary aim, we evaluated strain field homogeneity and strain stability and demonstrated negligible strain fluctuations over 18 h of cyclic stretching of membranes coated with fluorescent particles. We acquired live‐cell imaging data of cyclically stretched epithelial cells over a time span of 12 h and a temporal resolution of 50 s. Moreover, we demonstrated the possibility of switching between different stretch modes (uniaxial, biaxial, equibiaxial/radial) with automatic image acquisition within a single experiment.

Using the validated StretchView platform, we imaged MDCK epithelial cells in monolayer during stretching and evaluated the effect of uniaxial and radial cyclic stretching on cell kinematics, shape morphometrics, and the localization of the important cell‐cell adhesion protein E‐cadherin. Consistent with previous endpoint measurements of various cyclically stretched cell types, we discovered that cycle stretching leads to a gradual mild decrease in cell speed over the first 2 h of stretch, while speed recovers thereafter. Concurrently, cell elongation increases and shear rigidity decreases. Most importantly, we found that during the stretching period, cells in the monolayer monotonically increase their junctional E‐cadherin localization, which is suggestive of an increase in the barrier integrity of the monolayer. Although preliminary, these findings highlight the wealth of spatiotemporal information on the cells’ behavior that our platform can yield, and which can be further employed by the community to explore the role of stretching in regulating important cell processes such as motility and adhesion. Moreover, the particular preliminary findings are suggestive of stretch enhancing the barrier integrity of the monolayer, which might be a key finding if for example grafts of enhanced mechanical strength are desired for patient implantation but also in other aspects of tissue engineering.

Beyond the demonstrated functionalities of StretchView, the platformʼs largest advantages lie in its versatility. For example, by seeding cells on pre‐tensioned membranes,^[^ ^27^, ^71^ ^]^ cellular monolayers could also be compressed on the same platform without the need for design modifications. Similarly, the comparted actuation chambers that encircle the cell culture membrane enable the study of cells when stretched in various directions and axes. Additionally, more sophisticated strain waveforms can be applied (i.e., sinusoidal, trapezoidal, or waveforms derived from physiological measurements) via simple modifications to the pressure control script. By applying defined pressure ramps in trapezoidal waveforms, the overshoot response of the pressure control system that is caused by rectangular pressure signals can be addressed in the future, which enables stable vacuum levels at higher stretch frequencies (e.g., 1 Hz). The open‐top design facilitates membrane treatment (e.g., with adhesion promoters, and fluorescent particles), controlled cell seeding, and cell culture manipulation (e.g., via the introduction of growth factors, and pathogens). It additionally makes StretchView compatible with end‐point studies (e.g., western blotting, RNA extraction, immunostaining) as we demonstrated by fixation and subsequent staining of samples that were previously cyclically stretched with StretchView. Finally, microscopy and top access make StretchView compatible with other common biomechanics characterization methods such as traction force microscopy (TFM) and monolayer stress microscopy (MSM).^[^ ^72^ ^]^ This is an advantage over closed stretching systems such as organ‐on‐chip, where the controlled implementation of hydrogels (required for TFM and MSM) remains challenging.^[^ ^73^ ^]^

As with any custom tool that is fabricated by hand in a research laboratory, StretchView also comes with limitations that need to be taken into account, the biggest one of which is inter‐device variability. For example, small variations in the thickness or stiffness of the PDMS cell culture membrane could result in inconsistent vacuum‐strain characteristics and anisotropy of strain. Similar inconsistencies in performance can be caused by other device characteristics such as variations in the size of the actuation chambers, or slight warping in the 3D‐printed holders that can affect the vacuum interface. To a large extent, such limitations could be compensated by modification to the device design, calibration, and better control over the fabrication process, including the use of materials less likely to change over time. For example, a stiffer holder material that can withstand many incubations runs without geometrical warping can be applied. More practically, inter‐device stain variability and strain drifting during long experiments, for example due to the mechanical properties of PDMS changing during repeated deformation,^[^ ^74^ ^]^ could be compensated by automated vacuum adjustment controlled by the software. This approach necessitates real‐time monitoring of membrane strain during stretching experiments and requires the implementation of an algorithm that automatically performs DIC.

In summary, we believe the simple design and approachable fabrication and operation process of the StretchView platform, in combination with its unique and versatile set of features significantly lowers the barrier for the incorporation of stretching in cell culture and at the same time significantly increases the data yield of cell‐stretching experiments. Given that (extra)cellular physical forces, including stretching, play a determinant role in shaping biological processes, the possibility of studying cell‐ and tissue‐level behaviors in response to stretching on StretchView is expected to provide valuable insights into the dynamics of biomechanically regulated cellular processes.

## 4 Experimental Section

### Fabrication of the Cell‐Stretching System — *3D‐Printed Molds for PDMS Structuring*

The elastomeric device, including the device body and the stretching membrane, was made out of PDMS. The PDMS device body was made in molds fabricated using additive manufacturing, specifically using a stereolithographic 3D‐printer (Formlabs; Form 3). The used resin (Formlabs; High Temp V2) had a low heat deflection and the build platform allows for direct printing without the use of support structures (Formlabs; Build Platform 2). The STL geometry file was loaded into the preprocessing software (Formlabs; PreForm) and submitted to the 3D‐printer. Adaptive slicing layer height was applied to optimize printing time. The part was printed in such an orientation that the bottom surface of the mold lies flat on the build platform and the printed layers run parallel to the bottom surface. The printed molds were removed from the build platform by bending it, causing the 3D‐printed parts to detach. The molds were then washed upside down in isopropanol for 6 min in a washing device with a stirrer (Formlabs; Form Wash), rinsed with water, and dried with compressed air or nitrogen. The resulting green parts were subsequently placed in a post‐curing device (Formlabs; Form Cure) and were post‐cured for 120 min at 80 °C under UV light with a wavelength of 405 nm. Finally, the molds were coated with Parylene C by chemical vapor deposition inside a Parylene coating chamber (NTTF coatings; Smaland). This coating prevents chemicals leaching from the 3D‐printed molds that can inhibit the curing of PDMS as previously reported by Venzac et al.^[^ ^75^ ^]^ Additionally, the parylene coating serves as a non‐stick layer for better removal of PDMS.

### Fabrication of the Cell‐Stretching System — *PDMS Molding for Device Body*

PDMS (Dow; Sylgard 184) was mixed at a weight ratio of 10:1 (monomer to crosslinker) and degassed under vacuum. Molds were placed on a precision scale and 8.9 ± 0.1 g of PDMS were cast in each 3D‐printed mold and degassed under vacuum to remove trapped air bubbles. The filled molds were put in an oven for 3 h at a temperature of 80 °C, removed, and cooled down to room temperature. The casted PDMS parts were unmolded using a spatula. The vacuum interface channels were punched out using a 1 mm diameter biopsy punch.

For a schematic view of the device body fabrication, see Figure S7 (Supporting Information), steps a‐1 to a‐4.

### Fabrication of the Cell‐Stretching System — *PDMS Cell Culture Membrane Fabrication*

PDMS (Dow; Sylgard 184) was mixed at a weight ratio of 10:1 (monomer to crosslinker) and degassed under vacuum. The membrane was spin‐coated on a 4‐inch glass wafer (SPS; Polos Spin 150i). Using a repetitive pipette dispenser, we dosed 2 mL of PDMS on the center of the wafer and spun it for 60 s at a spin speed of 600 rpm. The membranes were cured on a hotplate at 80 °C for 10 min, removed, and cooled down to room temperature. This procedure resulted in a membrane thickness of approximately 100 µm (measured by Laser Scanning Microscopy of cut out edges). For a schematic view of the membrane fabrication, see Figure S7 (Supporting Information), steps b‐1 to b‐2.

### Fabrication of the Cell‐Stretching System — *Bonding of Device Body and Membrane*

The PDMS body and the membrane were bonded together by plasma‐activation (see Figure S7, Supporting Information, steps c‐1 to c‐4). The PDMS body and glass wafer carrying the membrane were exposed to oxygen plasma for 15 s at 100% power (Diener; Zepto BRS‐200). Immediately after plasma treatment, the PDMS device body part was placed on the membrane and gently pressed on it for a few seconds to promote bonding. The glass wafers now carrying the bonded device were then heated in an oven for 3 h at 60 °C and were then allowed to cool down to room temperature. With a scalpel, the membrane was cut out along the outer diameter of the device and was slowly peeled off the glass wafer.

### Fabrication of the Cell‐Stretching System — *Holder Parts and Spacer*

The device holder consisted of two halves. The holder parts and the spacer were additively manufactured using a material jetting 3D printer (Keyence; Agilista 3200W) using acrylic resin (Keyence; AR‐M2) and water‐soluble support material (Keyence; AR‐S1). The model geometries were imported as an STL file to the preprocessing software (Keyence; Modeling Studio), applying a printing layer height of 15 µm. The holder halves and the spacer were aligned so that the flat sides were flush with the building platform. The top holder parts were cleaned as follows: i) manual removal of the exposed support material with a spatula and wipes, ii) water bath for over 10 h, iii) blowing out internal channels with compressed air, iv) ultrasonic water bath for 10 min and v) repetition of step iii, vi) bath in absolute ethanol for 10 min, vii) washing with DI water and viii) drying with cleanroom wipes and compressed air. The bottom holder parts and the spacers were cleaned with steps i, iv, vi, vii, viii). The threads of the bottom holder were reinforced with stainless steel thread inserts (Boellhoff; Helicoil repair kit).

### Fabrication of the Cell‐Stretching System — *Assembly*

The cell‐stretching device was placed inside the holder. The bottom and top holder parts were screwed together with 4 metal screws. Subsequently, the spacer surface that is in touch with the membrane was lubricated by manually applying a thin layer of laboratory grease (Borer; Glisseal N). The spacer was then mounted on the holder top and fixed with 4 plastic screws. The opening of the cell culture well was covered with a cell‐culture dish lid (material: polystyrene, diameter: 35 mm) to prevent media evaporation and contamination.

### Operational Setup for Automated Cyclic Stretching

Figure S1A (Supporting Information) depicts a schematic view of the operational setup. For the vacuum control unit, a proportional pressure regulator valve (Festo; VEAB‐B‐26‐D18‐F‐V1‐1R1) was used and connected to a vacuum source comprising a vacuum nozzle (Festo; VN‐07‐H‐T2‐PQ1‐VQ1‐RQ1) for generating a vacuum from a compressed air supply (6 bar). The pressure regulator was controlled by analog output signals from a data acquisition card (NI; DAQ USB‐6001) connected to a PC via a USB cable. The analog voltage signal was controlled via Labview (using a modified version of the example file “Voltage – Continuous Output.vi”) or Matlab (applying the “Analog Output Generator” App). The pressure setpoint signal was set to a rectangular waveform with a period of 20 s, but could be adapted to arbitrary waveforms by changes in the Matlab script (e.g. sinusoidal or trapezoidal). The actual vacuum pressure (see Figure S1B for a schematic view and Figure S6, Supporting Information for an example measurement) was read out as an analog voltage signal with Labview (using the example file “Voltage – Continuous Input.vi”) or Matlab (applying the “Analog Input Recorder” App). The vacuum ports of the assembled stretching system were connected to the vacuum control unit via i) microfluidic connectors (IDEX H& S; flangeless nuts and ferrules, material: PPS), ii) microfluidic capillaries (outer diameter: 1/16′, inner diameter: 1.0 mm, material: PTFE), iii) a 3D‐printed adapter (Keyence; Agilista 3200 W, AR‐M2 resin), iv) pneumatic tubing (outer diameter: 4 mm, inner diameter: 2.5 mm, material: polyurethane) and v) pneumatic IQS standard push‐in‐fittings.

### Measurement and Analysis of the Pneumatic Response

For Figure S6 (Supporting Information), the sensor signal of the proportional pressure regulator was recorded by a data acquisition device (NI USB‐6001) and the Matlab application “Analog Input Recorder”. The recorded data (analog voltage signal) was further processed in OriginPro, including the application of the calibration factor to obtain the pressure signal and differentiation (central differences) for analysis of the pressure change rate over time. By a custom python script, peaks of the pressure change rate were extracted and the mean and SEM of peaks from n = 20 periods were calculated.

### Covalent Coating of PDMS with Fluorescent Tracer Particles

To measure the strains of the PDMS membranes, 0.5 µm‐diameter (for imaging with a 4x or 10x objective) or 0.2 µm‐diameter (for imaging with a 20x or 40x objective) fluorescent tracer particles were covalently attached (Thermofisher; F8811) to the surface of the PDMS membranes. First, the membrane was silanized for 3 min in ethanol with 5% APTES (3‐Aminopropyltriethoxysilane, Sigma; A3648), then washed 3 times with ethanol and dried in the oven for 30 min at 60 °C. The tracer particles (default ratio 1:100 and 1:50 for strain‐vacuum characteristics in Figure 3F) were diluted in 100 mm sodium hydrogen carbonate (NaHCO_3_) buffered in deionized water, then the solution was sonicated for 10 min and finally 600 µL of the obtained particle dilution was added to each elastic well. The dilution was left to settle down on the membrane for 5–10 min on a shaker. Afterward, the membranes were washed three times with deionized water, incubated in the above‐described NaHCO_3_ solution for 1 h on a shaker, then washed three times with deionized water and dried in an oven for 15 min at 60 °C.

### Cell Culture

Type II MDCK cells (generous gift from the Nelson lab, Stanford University) were cultured in high glucose DMEM medium (Thermofisher; 41965039) containing 4.5 g L^−1^ glucose and supplemented with 10% fetal bovine serum (FBS, Thermofisher; 10270106). Passages were between P10‐P50. Type II MDCK cells expressing E‐cadherin‐RFP were also a gift from the Nelson lab, Stanford University, and were cultured under the same conditions.^[^ ^76^ ^]^ During imaging, the cells were cultured in Leibovitz L‐15 medium without phenol red (Thermofisher; 21083027) containing 0.9 g L^−1^ galactose and supplemented with 10% FBS.

### Epithelial Cell Seeding, Fixation, Immunostaining, and Imaging

Before cell seeding, assembled stretching devices were UV sterilized for 1 h. The PDMS was then washed with phosphate buffer saline (PBS) and was then incubated with 100 µg mL^−1^ rat tail collagenΙ (Thermofisher; A1048301) in PBS for 2 h at 37 °C. Following a wash with PBS, 2×10^5^ MDCK cells were seeded on each PDMS membrane of the assembled StretchView device and incubated for 1 day at 37 °C, so that the cells formed a confluent monolayer prior to the initiation of stretching and imaging recording.

To test the effect of the lubricant Glisseal N on MDCK cells, Glisseal N was applied to the outer edge of wells of a glass‐bottom 24‐well plate (Cellvis), followed by UV sterilization for 1 h, and coating with 30 µg mL^−1^ rat‐tail collagen I at 37 °C for 30 min. The wells were washed once with PBS, 2×10^5^ MDCK cells/well expressing E‐cadherin‐RFP were seeded and incubated for 1 day at 37 °C. Then, cell nuclei were stained with 5 µg mL^−1^ Hoechst (Invitrogen) for 10 min, washed with PBS, and imaged with L‐15 medium supplemented with 5 µg mL^−1^ propidium iodide (PI, Thermofisher). To fix cells (on StretchView devices and well‐plates) the samples were carefully washed once with warm PBS, incubated with 4% methanol‐free paraformaldehyde (PFA, Thermofisher) for 10 min, washed again, and stored in PBS at 4 °C. For staining cells for F‐actin and immunostaining for Ki‐67, the fixed cells were permeabilized with 0.2% Triton‐X in PBS for 5 min, washed with PBS, and blocked with 5% BSA at room temperature for 30 min. Afterward, samples were incubated with fluorescently labeled phalloidin (1:100, Thermofisher, A12379) for 30 min or with the primary antibody Ki‐67 (diluted 1:100, Invitrogen, MA514520) in 2% BSA at room temperature for 1 h. Then samples treated with the primary antibody (Ki‐67) were washed three times with PBS for 5 min, blocked with 5% BSA for 30 min, incubated with the secondary antibody Alexa fluor‐647 (diluted 1:250, Thermofisher, A32795) for 1 h, again washed three times with PBS for 5 min and stored in PBS at 4 °C until imaging. Acquisition of z‐stacks of fixed immunostained cells on the StretchView device was performed using the Nikon Eclipse Ti2 spinning disk confocal microscope equipped with a CSU‐W1 spinning disc with 50 µm Disk and a digital camera C15440 (Hamamatsu), using the 20x/0.7 NA air objective, with a z‐spacing of 0.3 µm.

### Time‐Lapse Videomicroscopy of Epithelial Cells and Fluorescent Tracer Particles

Images were taken with an inverted microscope (Nikon; Eclipse Ti2) with a sCMOS camera (Teledyne Photometrics; Prime 95B) using an air objective with a magnification of 20x and a numerical aperture of 0.7 (Nikon; Super Plan Fluor LWD ADM) and the NIS‐Elements software package (RRID: SCR_014329). Images of cells and particles were acquired in separate channels. To maintain 37 °C during image acquisition, the microscope was surrounded by a box‐type incubator (OKOlab). For all experiments the period time of stretching was 20 s. The waveform of the vacuum was set to be rectangular (10 s in stretched and 10 s in relaxed state) in order to maintain a constant stretch magnitude during the image acquisition phases. Strain and direction of stretch varied depending on the experiment as indicated in the figures. Unless otherwise indicated, cells were recorded for at least 1 h before initiation of stretch and for at least 1h after cessation of stretch. The automatic acquisition of images was timed in such a way that the images of each stretching period were taken after a vacuum evacuation time of 3 s (in order to account for the vacuum stabilization time, as discussed in Section 2.6). The time interval between images was either 10 s, 30 s, 50 s, 2 min, or 10 min and images were acquired for up to 18 h. In detail: for the measurement of temporal strain stability in Figure 3G, the time interval for an image pair of the relaxed and stretched position was 10 s and image pairs were acquired every 10 min for a duration of 18 h; for the experiment with altering stretch configurations in Figure 3H, the time interval was 10 s for a total duration of 8000 s; for Figures 5, 6 and Figures S3, S4 (Supporting Information) the stationary condition was imaged every 50 s; for Figures 5, 6 the uniaxially stretched position was imaged every 2 min for the stationary periods and every 30 s for the stretched period; and for Figure 7 and Figure S5 (Supporting Information) the radially stretched condition was imaged every 50 s.

Live cell imaging of the Glisseal N treated cells in well‐plates (Figure S2, Supporting Information) was performed with the same microscope with the 40x/0.6 NA air objective. Images were taken every 10 min from 24 h to 45 h after seeding.

### Microscopy of Fluorescent Tracer Particles for Analysis of Strain‐Vacuum Characteristics

Tracer particles were applied on the membranes according to the protocol described in the section “Covalent Coating of PDMS with Fluorescent Tracer Particles”, applying a dilution ratio of 1:50 for the particle buffer dilution. Prior to imaging, the cell culture membranes were preconditioned by stretching for 60 cycles at 50% vacuum (0.5 bar). Image acquisition was performed manually under an inverted microscope (Olympus; IX73) with green LED illumination (CoolLED; pE‐300 white) and with a 4x magnification objective (Olympus; UPlanFL N 4x /0,13). Images were acquired in relaxed states and stretched states with vacuum percentages from 5 to 50% in steps of 5% (0.95 down to 0.5 bar in steps of 0.05 bar).

### Digital Image Correlation and Evaluation of Strain

For DIC, the software MATLAB (Mathworks) together with PIVlab was used.^[^ ^77^ ^]^ Image sequences for analysis were first prepared in Fiji/ImageJ.^[^ ^78^ ^]^ These preparations included the import of image stacks in TIFF or ND file format, followed by adjustment of contrast and brightness, de‐interleave, and export of a series of individual image files in PNG file format. The image series was then imported to PIVlab using the pairwise image sequencing style (represented by the pattern “A+B, C+D”), which means that each image of the stretched state (respectively “B” and “D”) is referred to the image of the unstretched within its own stretch period (respectively “A” and “C”). In other words, the unstretched reference image was updated at each stretch period (from “A” to “C”). The image data was preprocessed using CLAHE (Contrast Limited Adaptive Histogram Equalization) with a window size of 64 px. The region of interest (ROI) was selected and the calibration value was set according to the calibration factor of the image acquisition. As the aim was evaluating displacements instead of velocities, a (pseudo‐)time step size of 1 s was applied in order to have a velocity vector field that equals the displacement vector field. As PIV algorithm, we chose “FFT window deformation” and applied interrogation areas of 320 px with a 160 px step in Pass 1, 160 px (80 px step) in Pass 2, and 80 px (40 px step) in Pass 3. The obtained results, containing the displacement vector field for each time frame, were exported as text files. Subsequently, a custom python script was used for the calculation of Lagrange strains and calculation of the average strain magnitudes. The Lagrange strain tensor ***E*** is defined as:

(1) $$E\;=\;\frac{1}{2}\left[grad{\left(U\right)}^{T}grad\left(U\right)\;-1\right]$$

where ***U*** is the displacement vector and 1 is the second‐rank identity tensor. Average strains in xx, yy, and xy directions were calculated by the mean of strain field matrix entries for each time frame. Normalized average strains were calculated by dividing the average strains of each time frame by the strain at t = 0 min. The Green‐Lagrange strain field matrix was cropped at the borders of the region of interest in order to ignore boundary artifacts that are inherent to the DIC process. Defocused regions that could not be evaluated properly and resulted in inhomogeneous/noisy strain data were also cropped and not considered for further strain analysis.

### Evaluation of Cell Kinematics and Junctional E‐Cadherin Abundance

To quantify cell kinematics and calculate mean cell speed, an image correlation technique similar to particle image velocimetry was used,^[^ ^49^ ^]^ which was applied on the phase contrast images of cells as done previously using Matlab (Mathworks).^[^ ^51^, ^79^ ^]^ The codes can also be found here: https://github.com/ebastoun/Correlation_length_of_movement_of_epithelial_cells_in_monolayer. Subsequent frames of cells were compared under stationary conditions at a 2 min time interval using interrogation windows of 32 × 16 px (window size x overlap). In the case of cells cyclically stretched, successive frames were compared in the 2‐min‐interval between each unstretched configuration and the subsequent one. Mean cell speed was calculated as the average cell displacement at each FOV divided by the frame interval.

To systematically characterize temporal alterations in pericellular E‐cadherin localization of cells subjected to stretch, timelapse epifluorescence imaging of type II MDCK cells expressing E‐cadherin‐RFP was conducted. The resulting epifluorescence images were first background subtracted using ImageJ (RRID:SCR_002285) and a rolling disk of 100 px. The background‐subtracted E‐cadherin fluorescence images were then read in MATLAB, and the integral of E‐cadherin fluorescence was calculated as a function of time.

### Quantification of Cell Shape Characteristics during Cell‐Stretching

Single cell tracking and characterization of cell‐based morphological parameters were performed using the ImageJ plugin Trackmate V7 on the phase contrast image of cells (Movies S5 and S6).^[^ ^59^ ^]^ Specifically, (aligned) stretched and unstretched images were first registered with respect to a specific point in the image corresponding to an intersection between cells and being close to the center of the original phase contrast images. After image registration, images centered with respect to that point were cropped in smaller segments (165 µm x 165 µm) containing ≈70 cells. Then these images of cells under stretch (or not) were loaded in Trackmate, and the Cellpose segmentor was used, which is based on a deep learning segmentation method that does not require model retraining.^[^ ^60^ ^]^ Segmented cells were then tracked over time, and cell‐specific parameters were exported, and imported in MATLAB for further analysis. Using additional custom‐written code, the cell area (in µm^2^) and the cell circularity was calculated, which is defined as $4\frac{\pi \cdot area}{perimeter^{2}}$ and is a measure of how close to a circle the shape of a given cell is. If a cell resembles a nearly perfect circle, then circularity should be close to 1. On the contrary, this parameter will get closer to zero for more elongated cells. The shape factor (also referred to as shape index or shape parameter) was also calculated which is defined as the cell $\frac{perimeter}{\sqrt{area}}$ and can be thought of as a measure of shear rigidity with isotropic solid‐like cells having a shape factor close to the critical value of 3.81 and more fluid‐like cells (reduced fluid rigidity) having a higher value.^[^ ^51^, ^62^ ^]^ The above parameters were examined on a per‐cell basis during stretch. For each cell, averages were calculated by normalization with respect to the value of the respective parameter at the first‐time frame of the recording. Additionally, for a given recording we also calculated the mean value of all cells for a given parameter and plotted it over the course of time.

### Statistical Analysis

For the case of experiments involving live‐cell microscopy imaging, after image processing was performed, statistical analysis was done using Graphpad Prism 10 (Graphpad, RRID:SCR_002798). For comparing two distributions, sina plots superimposed with the mean and standard deviation (SD) of the distributions were used (e.g., Figure S2D, Supporting Information). In all cases, data originating from at least three independent recordings (i.e., wells) were analyzed. The corresponding figure legends indicate statistical tests performed, p‐values, and sample size. For the comparison of two distributions, the non‐parametric Wilcoxon Ranksum test (denoted as WRST) was used which does not assume the normality of the data.

For the measurement of strain‐vacuum characteristics (Figure 3F), independent measurements were carried out n = 3 times per stretch mode, using the same device. Linear regression was carried out in OriginPro software. The figure legend indicates the standard deviation and linear regression.

For the measurements of temporal strain stability (Figure 3G) and for the experiment with switching stretch modes (Figure 3H), averaged strains for each time point were obtained by the spatial average over a region of interest of 500 × 500 µm, resolved by 484 strain data points (Figure 3G) and 400 × 400 µm, resolved by 306 strain data points (Figure 3H). Further information on data processing for DIC is given in Section “Analysis of Imaging Data” of the Experimental Section.

In the analysis of pneumatic response characteristics (Figure S6, Supporting Information), n = 20 peaks were evaluated for the evaluation of pressure change rates during the application of stretch and relaxation respectively. Mean values and SEM are indicated in the figure legends. Further information is given in Section “Operational Setup for Automated Cyclic Stretching” under the subhead “Measurement and Analysis of the Pneumatic Response” in the Experimental Section.

## Conflict of Interest

The authors declare no conflict of interest.

## Author Contributions

E.B. and I.C. conceived the work and provided input into the StretchView device design and experimental design. D.J. developed the StretchView platform and related characterization methodologies and operation processes. L.H. performed experiments that involved cells. L.H. and E.B. performed an analysis of experimental results related to cell experiments. E.B. and I.C. acquired funding, supervised experimental work and data analysis, and administered the project. D.J., L.H., E.B., and I.C. co‐wrote the manuscript and participated in review and editing.

## Supporting information

Supporting Information

Supporting Information

Supporting Information

Supporting Information

Supporting Information

Supporting Information

Supporting Information

Supporting Information

## Data Availability

The data that support the findings of this study are available from the corresponding author upon reasonable request.
