# Supplementary material for: StretchView – A Multi‐Axial Cell‐Stretching Device for Long‐Term Automated Videomicroscopy of Living Cells
Source: Adv Sci (Weinh). 2025 Jan 10;12(9):2408853. doi: 10.1002/advs.202408853 (PMC11884571; doi:10.1002/advs.202408853)
Supplement: Supplementary file 1 — Supporting Information [file ADVS-12-2408853-s001.docx]

# Supporting Information

StretchView - a multi-axial cell-stretching device for long-term automated videomicroscopy of living cells

*David Jaworski^1,2^, Lara Hundsdorfer^3,4^, Effie Bastounis^3,4,^*, Iordania Constantinou^1.2,^**

^1^ Institute of Microtechnology (IMT), Technische Universität Braunschweig, Alte Salzdahlumer Str. 203, 38124 Braunschweig, Germany

^2^ Center of Pharmaceutical Engineering (PVZ), Technische Universität Braunschweig, Franz-Liszt-Str. 35a, 38106 Braunschweig, Germany

^3^ Interfaculty Institute of Microbiology and Infection Medicine (IMIT), University of Tübingen, Auf der Morgenstelle 28, 72076 Tübingen, Germany

^4^ Cluster of Excellence "Controlling Microbes to Fight Infections" (CMFI), EXC 2124, University of Tübingen, Auf der Morgenstelle 28, 72076 Tübingen, Germany

E-mails: i.constantinou@tu-braunschweig.de, [effie.bastounis@uni-tuebingen.de](mailto:effie.bastounis@uni-tuebingen.de)


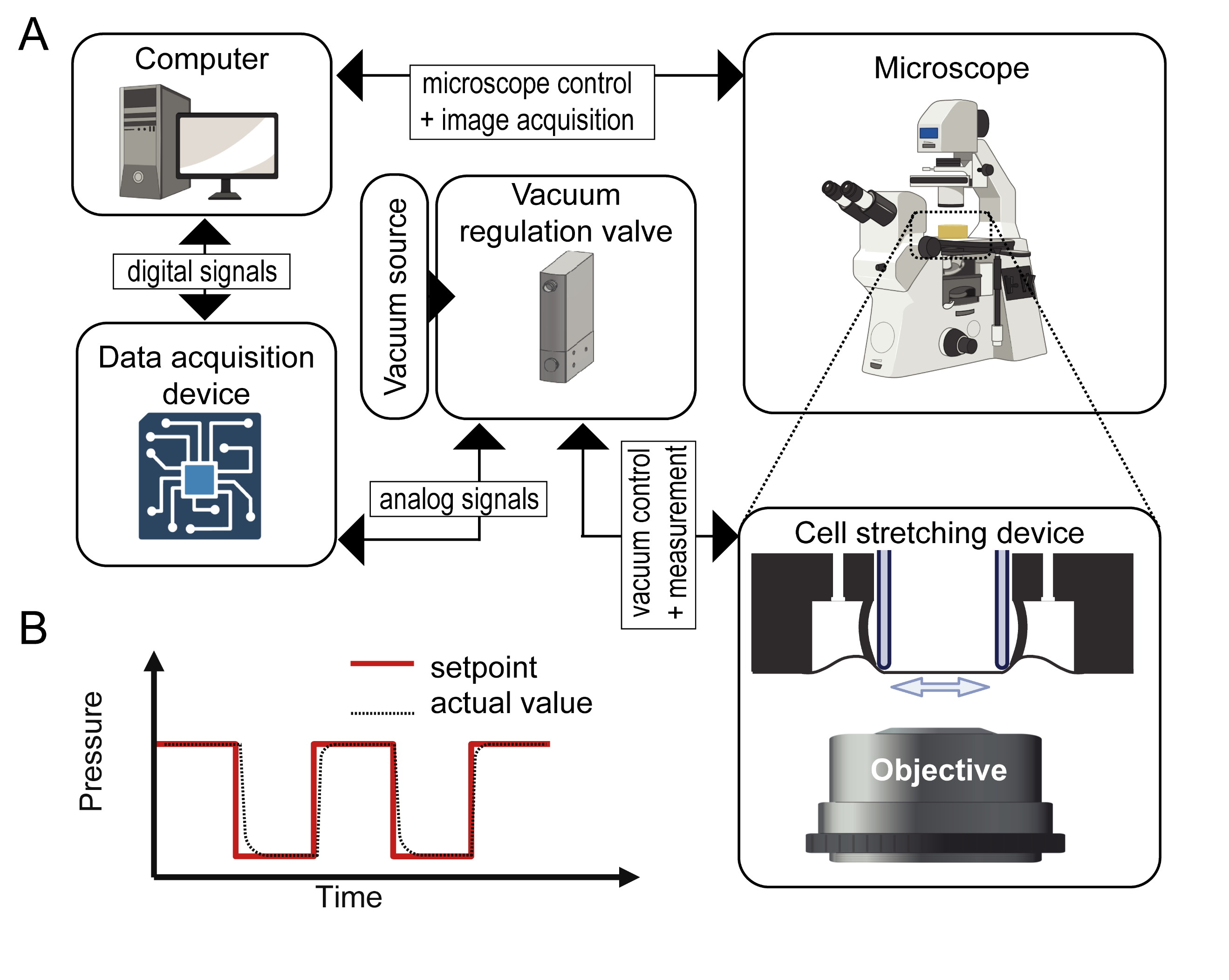


**Figure S1.** Control setup for live-cell imaging of stretching cells. (A) The setup includes a microscope with a cell stretching device, computer, control valves, a data acquisition device (DAQ) and a voltage and vacuum supply. The cell stretching device is implemented into the inverted microscope via a 3D-printed holder in well plate format. The vacuum ports on the holder of the cell stretching device are connected to one or more proportional pressure control valves which regulate the pressure from the vacuum supply. The periodic vacuum pressure signal (setpoint) is generated digitally via a computer in the Matlab or Labview software and converted into an analog voltage signal with the DAQ, which controls the proportional pressure control valve. At the same time, the actual value of the vacuum pressure is measured by the proportional pressure control valve and sent out as an analog voltage value, to the DAQ, which transmits it as a digital signal to the computer. (B) Schematic of the rectangular waveform of the periodic setpoint function and the actual value which has a delay due to evacuation time of the vacuum chambers. Figure created with BioRender.com.


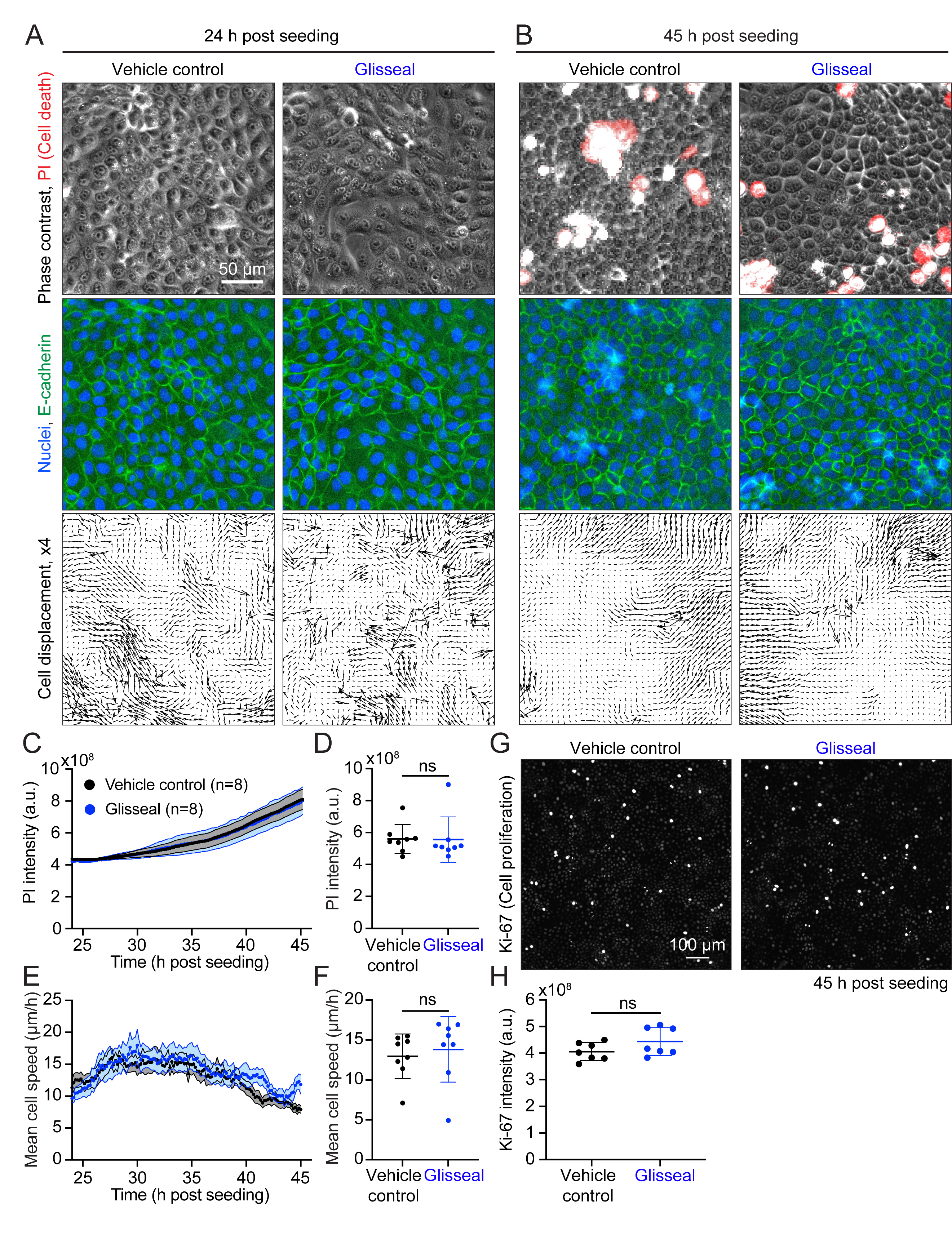


**Figure S2.** Presence of Glisseal N does not alter MDCK cell motility, proliferation, apoptosis, nor intercellular adhesion organization. (A-B) Representative time-lapse microscopy images of MDCK cells in monolayer expressing E-cadherin-GFP under stationary conditions in the absence (vehicle control, 1^st^ column) or presence of Glisseal N (Glisseal, 2^nd^ column) at 24 h (A) and at 45 h post-seeding (B). For the purposes of this comparison cells were seeded on collagen I-coated glass coverslips. Imaging was performed from 24 to 45 h post-seeding with a frame interval of 10 min. Rows show: phase contrast image (grayscale) superimposed with propidium iodide (PI) fluorescence to mark dead cells (red); Hoechst-dyed cell nuclei (blue) image superimposed with E-cadherin fluorescence (green); and cell displacement vectors (black arrows) scaled 4x. Scale bar: 50 μm. (C) Plot of integral (sum) of PI fluorescence intensity (a.u.) for vehicle control (black) or Glisseal N exposed (blue) samples over time. N=8 independent microscopy recordings were performed originating from independent wells but experiment was performed at the same day. Mean +/- SEM. (D) Mean integral (sum) of PI fluorescence intensity per replicate from panel C. Mean +/- SD. WRST: ns: non-significant. (E) Same as panel C but showing mean cell speed over time as determined via PIV on the phase contrast image of cells. (F) Mean cell speed per replicate from panel E. Mean +/- SD. WRST: ns: non-significant. (G) Representative examples of immunostained for Ki-67 samples (cell proliferation marker) 45 h post-seeding for vehicle control samples (left) or Glisseal N exposed (right). (H) Quantification of the integral (sum) of Ki-67 fluorescence intensity (cell proliferation marker) for different fields of view imaged (N=7) for vehicle control samples (left) or Glisseal N exposed (right) at 45 h post seeding. Lines indicate mean±SD, WRST: ns: non-significant.


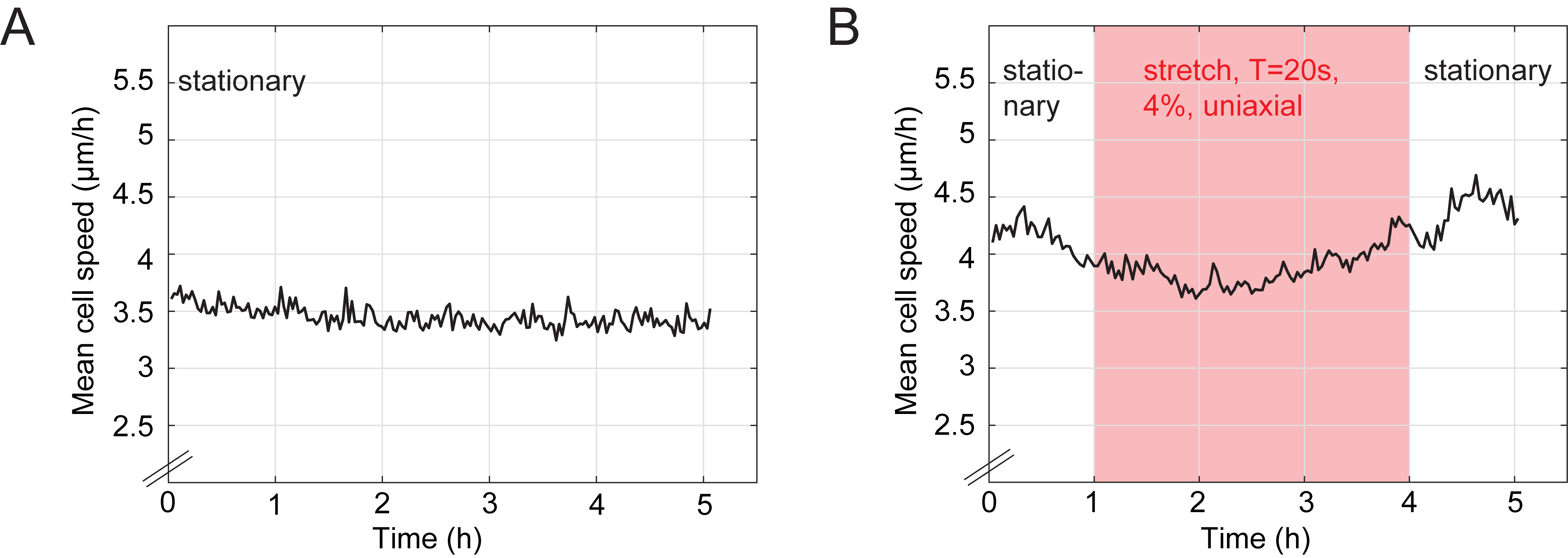


**Figure S3.** (A) Plot of mean cell speed (μm/h) versus time (h) for the representative example of cells under stationary conditions shown in Figure 5A. (B) Same plot as in panel A but referring to the representative acquisition shown in Figure 5B. Note that in the first and last hour of the 5-h long acquisition, cells were under stationary conditions, and were only subjected to 4% uniaxial stretch with T = 20 s from t = 1 – 4 h (see red shaded area).


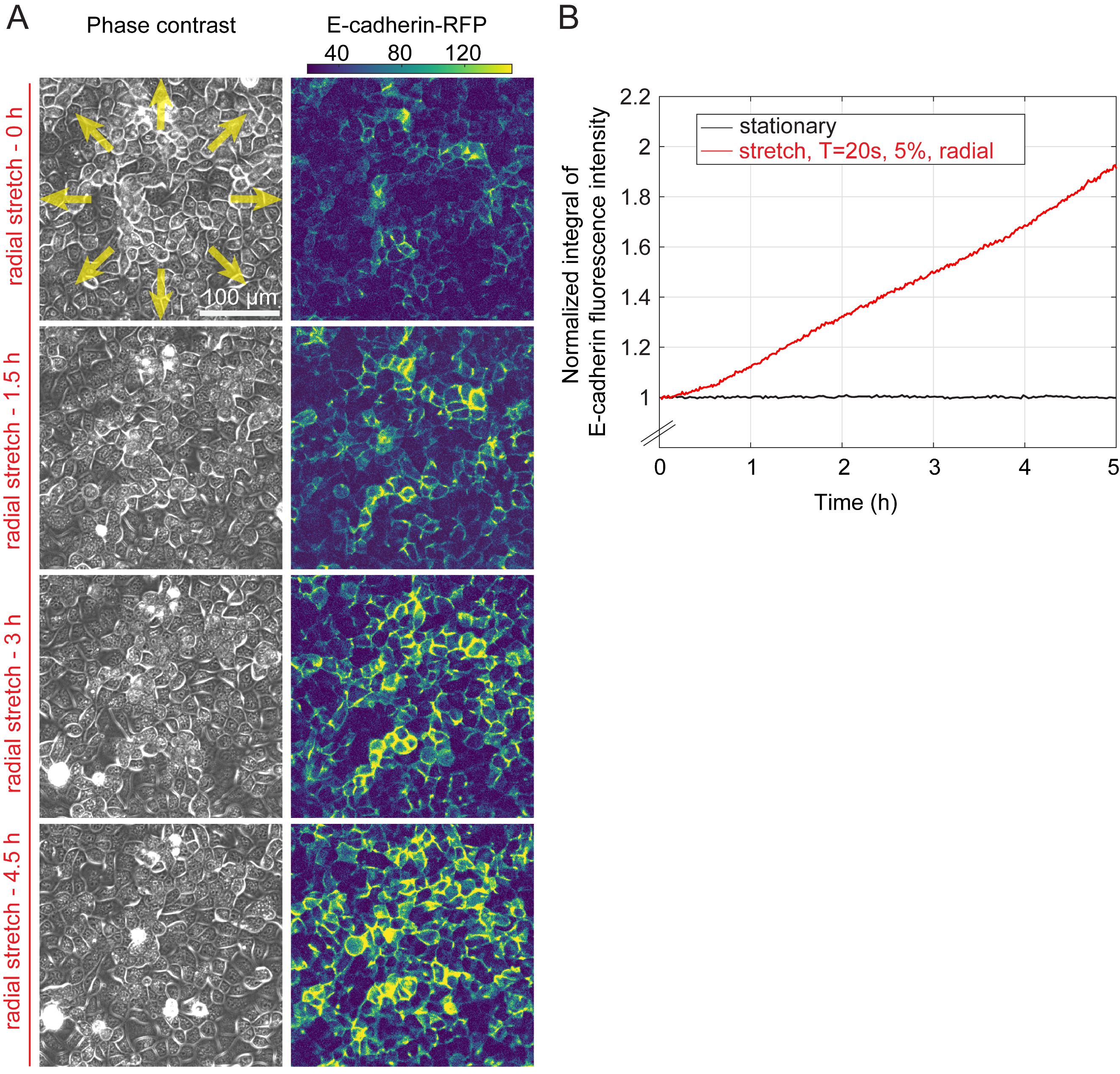


**Figure S4.** Epithelial cells show a monotonic increase in the localization of E-cadherin at cell-cell junctions also when subject to periodic radial stretch. (A) Representative time-lapse epifluorescence microscopy images of MDCK cells in monolayer constitutively expressing E-cadherin-RFP when continuously subject to 5% radial with T = 20 s (see yellow arrows showing direction of stretch). Columns show phase contrast image (1^st^ column) and corresponding background-corrected E-cadherin fluorescence intensity image (2^nd^ column). Rows show 4 different time points along the time-lapse acquisition. Note that subsequent phase contrast images of cells in the “unstretched” configuration were compared and that the frame interval used for the comparisons was 2 min. (B) Plot of normalized integral of E-cadherin fluorescence intensity versus time (h) for the representative example of cells under radial stretch shown in panel A (red) as well as the control cells under stationary conditions shown in Figure 6B (black). Integration was performed over the whole FOV imaged, and normalization was performed with respect to the first image of the acquisition, so that differences in fluorescence intensity overtime became apparent. See also Figure 6.


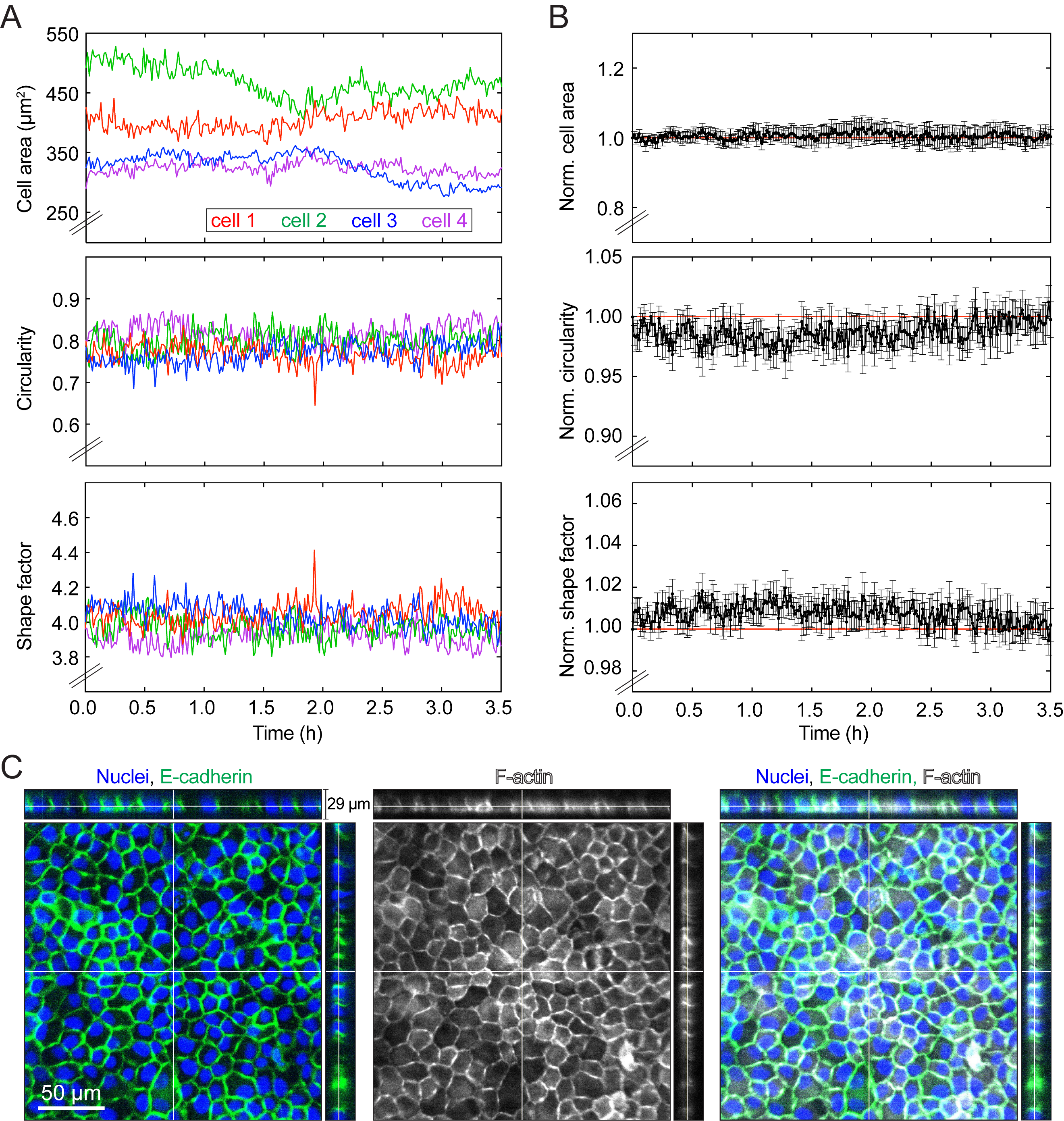


**Figure S5.** Epithelial MDCK cells under stationary conditions show a constant area, shape factor, and circularity over the course of time. (A) Upper plot shows cell area (μm^2^) versus time of four tracked cells. Middle plot shows the circularity versus time of the same four cells and bottom plot shows their shape factor versus time. (B) Plot of normalized mean cell area (upper), circularity (middle) and shape factor (bottom) versus time for N=11 individually tracked cells. Values for each of the three parameters of each cell were normalized relative to the value at t=0 s (first frame from time-lapse recording). Vertical bars show standard error of the mean (SEM). (C) Orthogonal views of fixed MDCK cells previously stretched biaxially (8% strain in x- and 2% strain in y-direction) with T= 20 s for 22 h. Left image shows overlay of MDCK cell nuclei (blue) and E-cadherin fluorescence (green), middle image shows F-actin (staining done using phalloidin), and right image shows the overlay of all three aforementioned signals. Scale bar is 50 μm. One representative example is shown as a proof-of-concept that fixation followed by (immuno)staining of samples previously stretched using StretchView is possible.


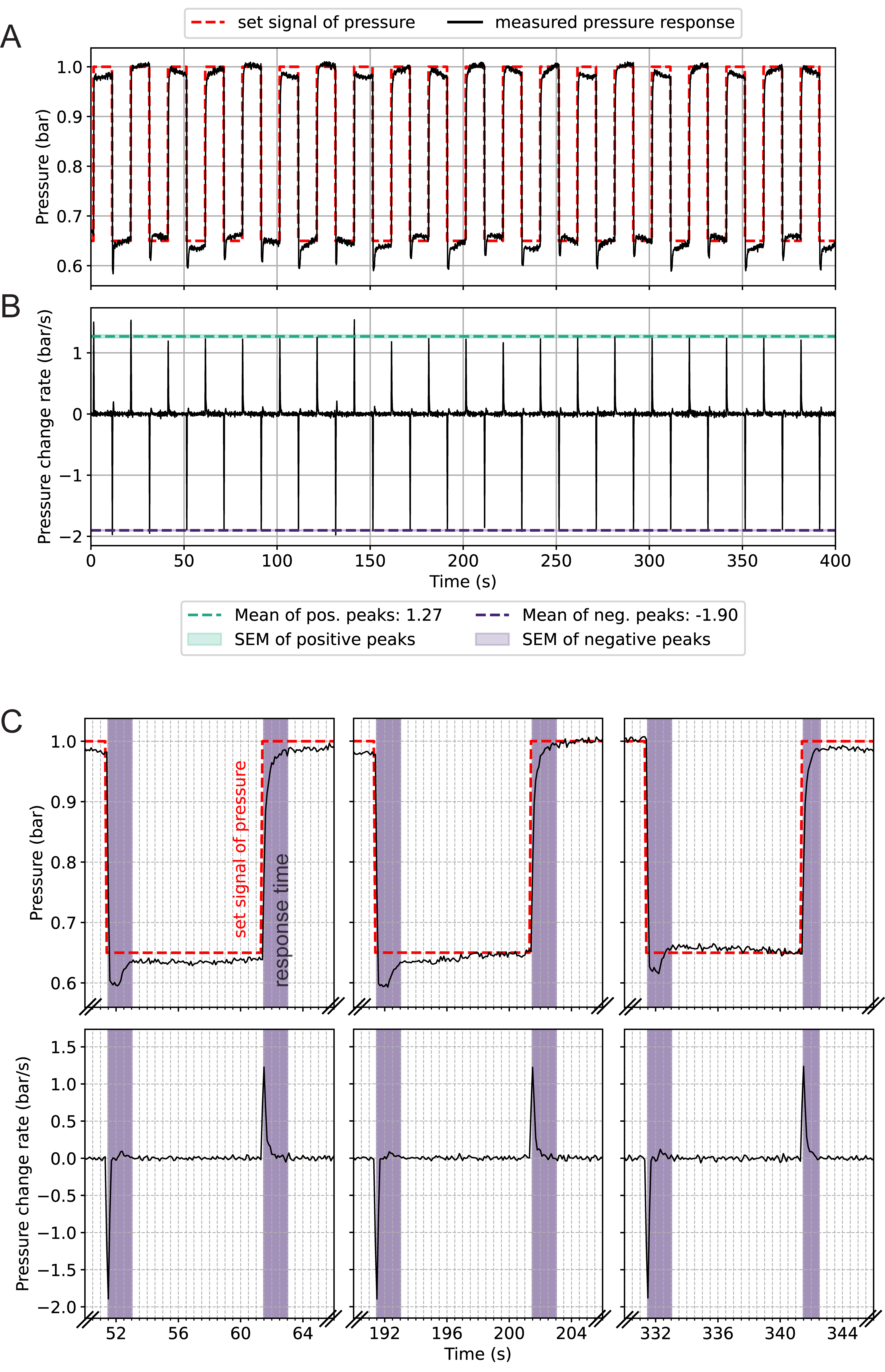

**Figure S6**. Measured pressure response over time upon actuation of StretchView. (A) Actual pressure signal measurement over 20 stretch periods at the proportional pressure regulator valve for a set pressure signal (red) defined by a rectangular waveform with 20 s period time and a vacuum level of 35%. Note that the measurement signal has an inherent waviness from the pressure valve sensor. The measured signal exhibits an overshoot response due to the steep applied pressure ramp based on the rectangular waveform of the pressure set signal. (B) Temporal derivation of the pressure signal yields the pressure change rate over time (bar s^-1^). The pressure change rate peaks during stretch and relaxation (i.e., application of vacuum and return to atmospheric pressure). The mean pressure change rate peaks (over n=20 periods) are plotted as dashed green and purple lines (SEM highlighted as transparent area). The mean pressure change rate has a magnitude of 1.9 bar s^-1^ and 1.3 bar s^-1^ for application of stretch and for relaxation, respectively. The data was acquired in the experiment associated to Figure 3H during radial stretch at 8% strain. Correlation with measured pressure change rate yields estimated strain rates of 46 and 30 % s^-1^ for stretching and relaxation, respectively. (C) Detailed views of pressure and pressure change rate over time for three selected time intervals. The set pressure signal is indicated in dashed red lines and the qualitatively observed response time for stabilization of the pressure is highlighted in purple shaded time intervals. During the experiment we majorly observed a response time of 1.5 s, which was considered in the timing of image acquisition.

**
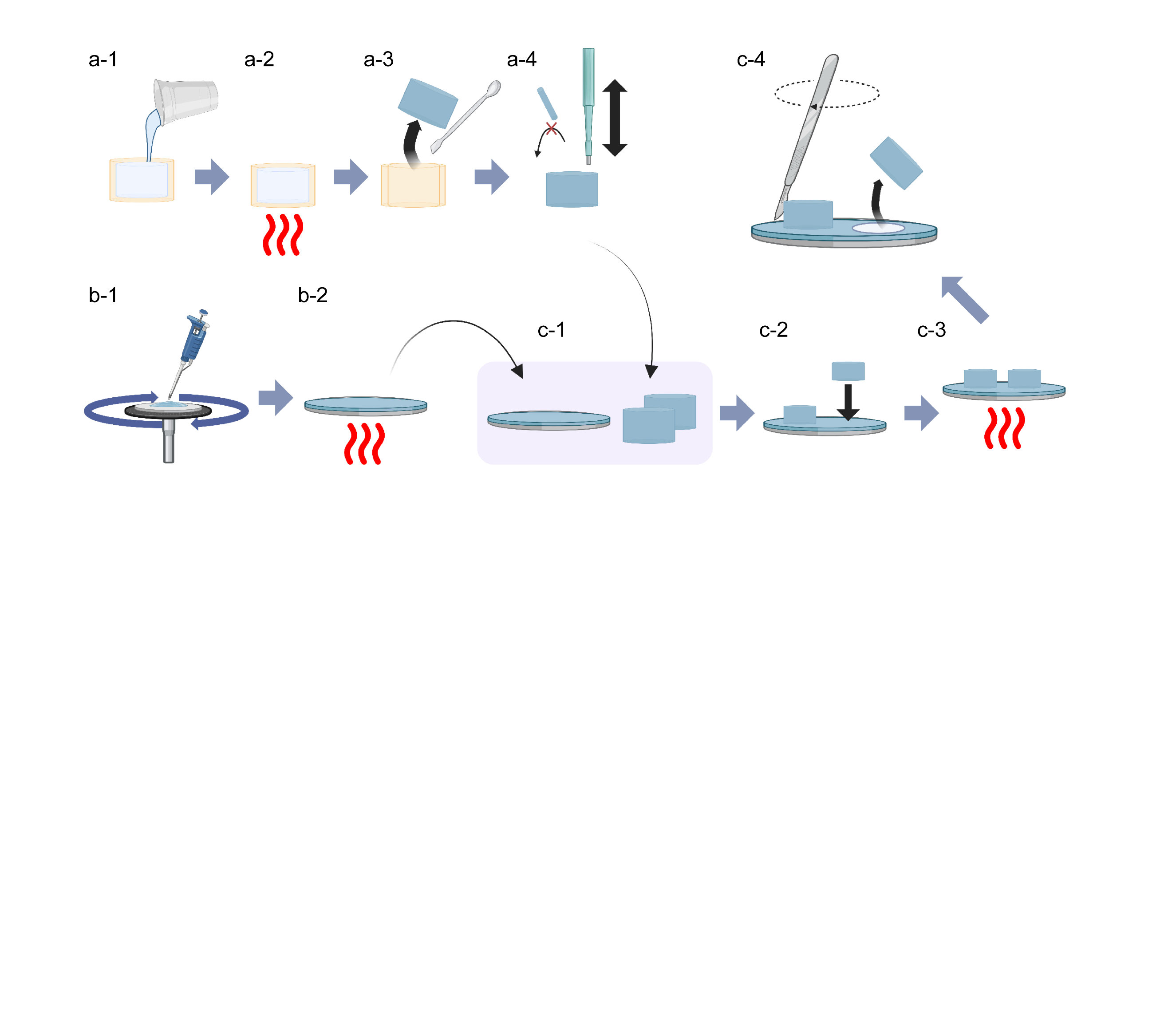
**

**Figure S7**. Manufacturing steps of the cell stretching device. The prepared PDMS mixture is poured into the 3D-printed mold (a-1) and cured in an oven (a-2). Then, the cured PDMS is gently removed with a spatula (a-3) and the channels for the interface with the comparted vacuum chambers are punched out with a biopsy punch (a-4). The PDMS membrane is spin-coated on a glass wafer (b-1) and cured on a hotplate (b-2). For plasma bonding, the PDMS membrane and the molded PDMS parts are first treated in O2 plasma (c-1). After removal from the plasma chamber, the molded PDMS part is placed perpendicularly on the PDMS membrane and the parts are gently pushed together (c-2). Then the bonded parts are heated in an oven (c-3). Finally, the devices are cut out with a scalpel and gently peeled off by hand (c-4). Figure created with BioRender.com.

**Supporting Movies**

All Supporting Movies can be downloaded at <https://doi.org/10.24355/dbbs.084-202410280821-0>. Below movie thumbnails with the respective captions are provided.


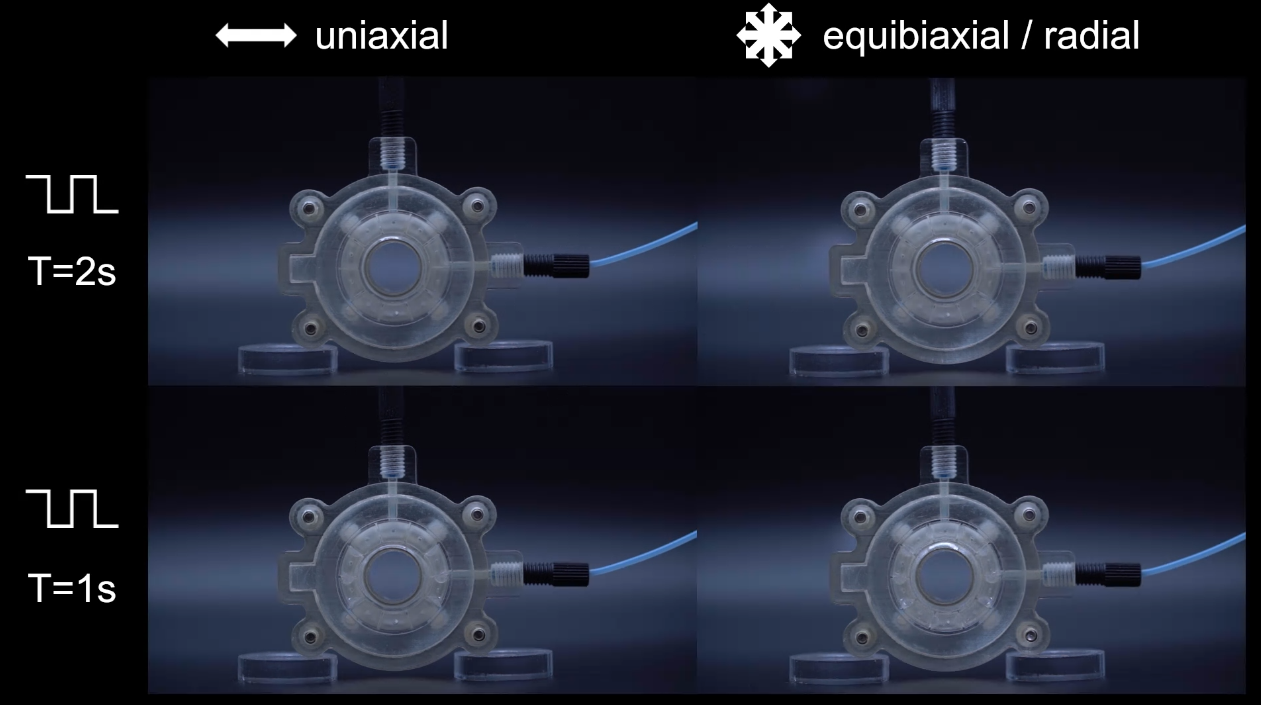


**Movie S1.** Videos of the StretchView device under actuation. The device without medium was placed laterally on a table to capture the contracting actuation chambers. The first half of the video shows actuation with a rectangular waveform signal and the second half shows actuation with sinusoidal waveform signal. Left column: uniaxial stretch; right column: radial (equibiaxial) stretch; top row: period time of 2 s; bottom row: period time of 1 s.

**[
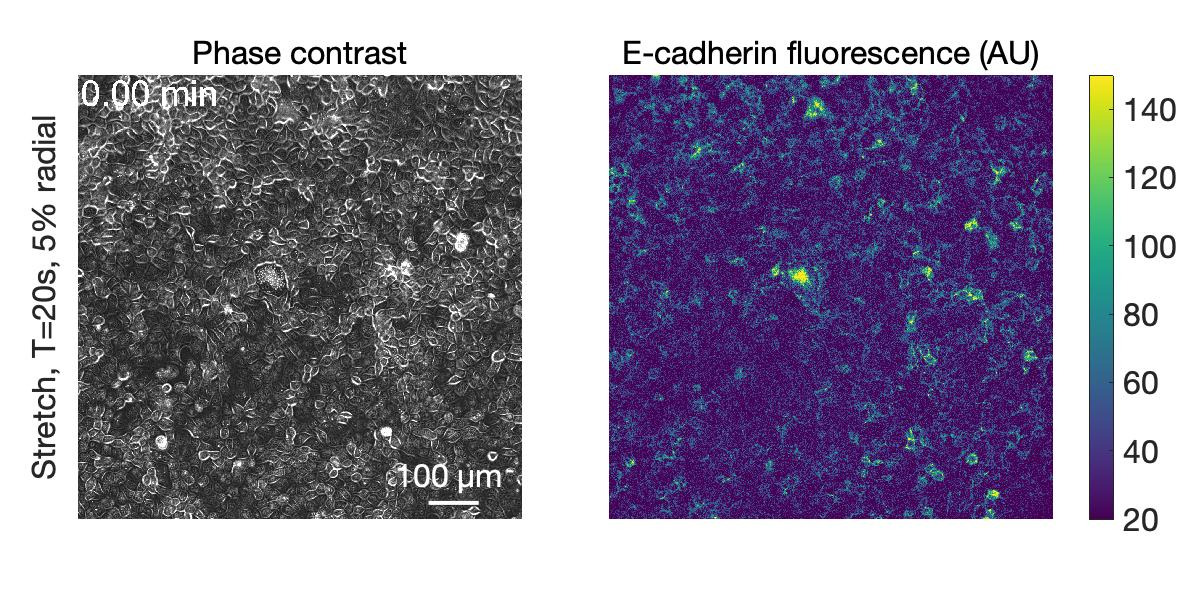
](https://doi.org/10.24355/dbbs.084-202410280821-0)**

**Movie S2**. Movie of a representative time-lapse microscopy recording of radially stretched MDCK epithelial cells in monolayer imaged over the course of 12 h. Stretched and stationary state are shown alternating. Left panel shows the phase contrast image of MDCK cells constitutively expressing E-cadherin-RFP under conditions of 5% radial stretch with a period of T=20 s. The time post-initiation of recording is indicated in minutes on the top left corner and scale bar is 100 μm. Specifically, every 50 s a phase contrast image and an image of the E-cadherin fluorescence of first the unstretched and subsequent stretched configuration were acquired. The right panel shows the corresponding background subtracted E-cadherin fluorescence intensity image. For clarity and to avoid creating an overly large movie file, we present images at 650 s intervals (10 min). Same data as in Figure S4 and Movie S5, but whole field of view and longer imaging period shown, demonstrating that automated long-term microscopy of stretched cells is possible.

[
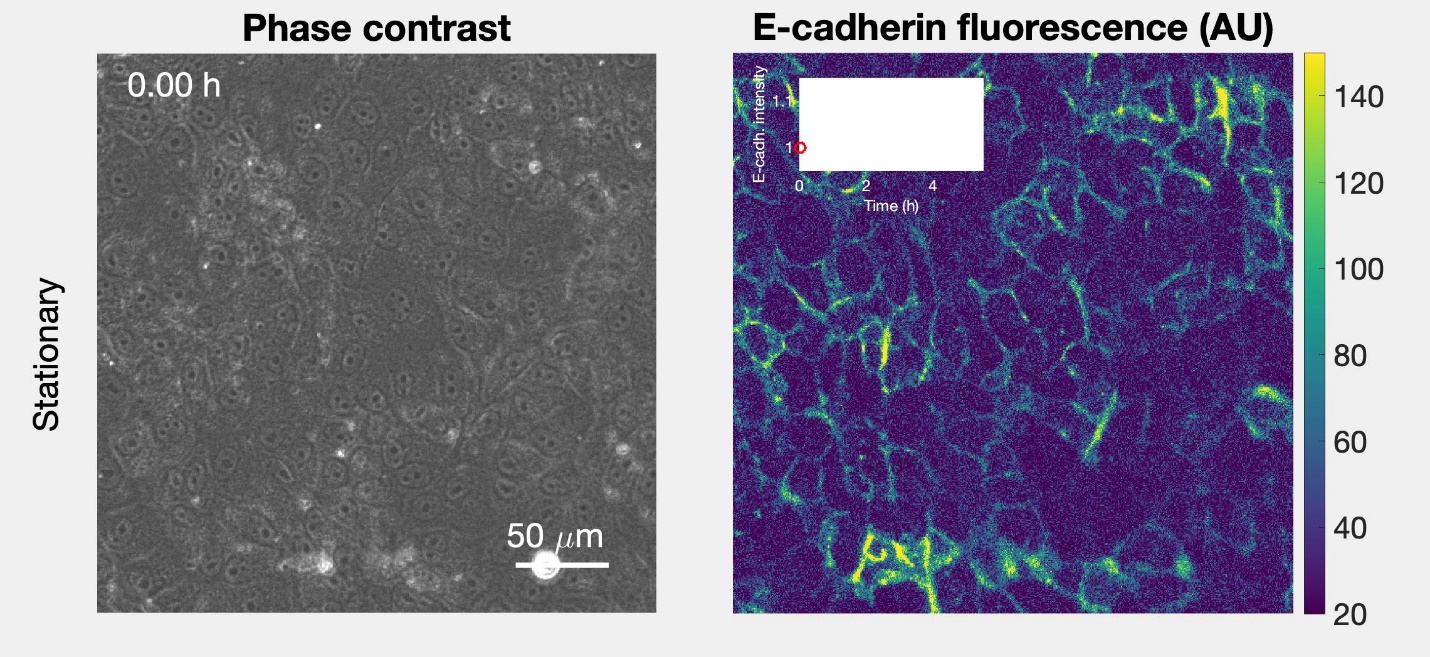
](https://doi.org/10.24355/dbbs.084-202410280821-0)

**Movie S3.** Time-lapse movie of confluent epithelial cells under stationary conditions showing no fluctuations in the localization of E-cadherin at cell-cell junctions. Left panel shows the phase contrast image of MDCK cells in monolayer constitutively expressing E-cadherin-RFP under stationary condition (no stretch). The time is indicated in hours on the top left corner and scale bar is 50 μm. The right panel shows the corresponding background subtracted E-cadherin fluorescence intensity. The superimposed plot on top left corner shows the integral (sum) of the E-cadherin fluorescence intensity as a function of time. The value referring to the frame shown is indicated by a red circle.

[
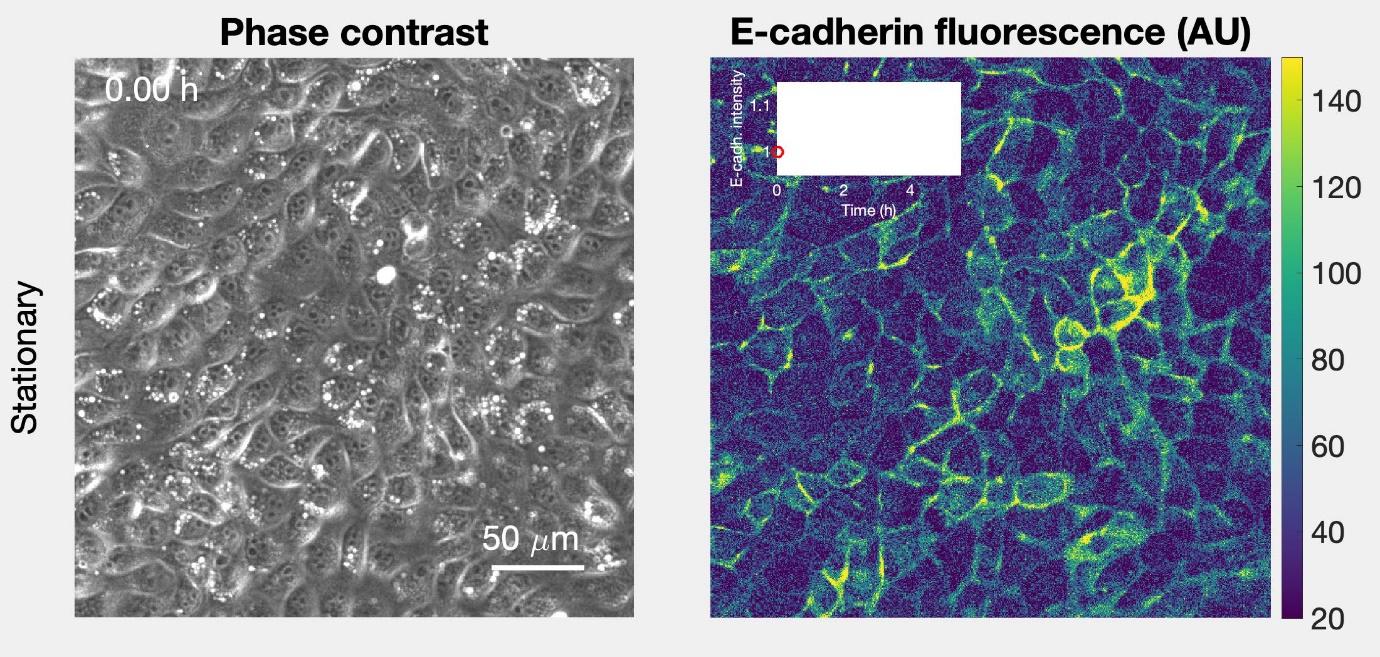
](https://doi.org/10.24355/dbbs.084-202410280821-0)

**Movie S4.** Time-lapse movie of epithelial cells in monolayer subject to uniaxial stretch show a progressive enhancement in the localization of E-cadherin at cell-cell junctions. Left panel shows the phase contrast image of MDCK cells in monolayer constitutively expressing E-cadherin-RFP under stationary conditions (0 - 1 h) followed by application of 5% uniaxial stretch with a period of T=20 s (1 - 4 h) and then a pause of stretching (4-5 h). The vertical legend on left side of panel indicates whether cells are stretched or not. The time post-initiation of recording is indicated in hours on the top left corner and scale bar is 50 μm. The right panel shows the corresponding background subtracted E-cadherin fluorescence intensity. The superimposed plot on top left corner shows the integral (sum) of the E-cadherin fluorescence intensity as a function of time. The value referring to the frame shown is indicated by a red circle. Note the quasi-linear reinforcement of pericellular E-cadherin fluorescence intensity during application of stretch.

**[
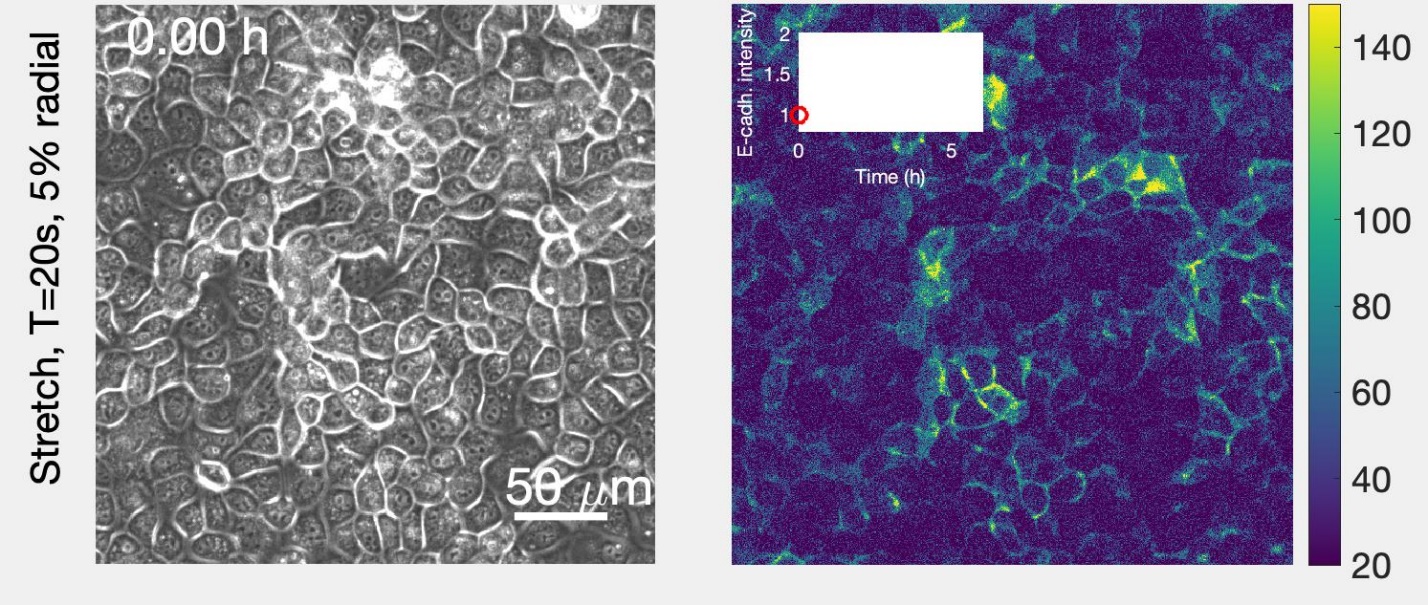
](https://doi.org/10.24355/dbbs.084-202410280821-0)**

**Movie S5.** Time-lapse movie of epithelial cells in monolayer subject to radial stretch show also linear increase in the localization of E-cadherin at cell-cell junctions. Left panel shows the phase contrast image of MDCK cells in monolayer constitutively expressing E-cadherin-RFP under conditions of 5% radial stretch with a period of T=20 s. The time post-initiation of recording is indicated in hours on the top left corner and scale bar is 50 μm. The right panel shows the corresponding background subtracted E-cadherin fluorescence intensity. The superimposed plot on the top left corner shows the integral (sum) of the E-cadherin fluorescence intensity as a function of time. The value referring to the frame shown is indicated by a red circle. Note the quasi-linear reinforcement of pericellular E-cadherin fluorescence intensity during application of stretch.

[
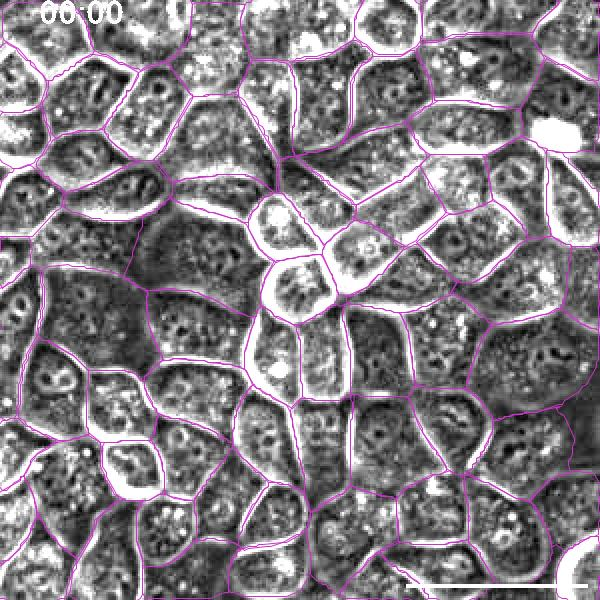
](https://doi.org/10.24355/dbbs.084-202410280821-0)

**Movie S6.** Phase contrast image and segmentation of radially stretched epithelial cells in monolayer with overlayed cell contour in magenta. The scale bar corresponds to 50 μm. Time-lapse movie showing the phase contrast image of MDCK cells subject to 5% radial cyclic stretch. Period of stretching is 20 s and the frame interval displayed is 30 s. Superimposed contours in different colors show the cell outlines detected using Cellpose detector of ImageJ plugin Trackmate. On the upper left corner, the time is indicated in min.

[
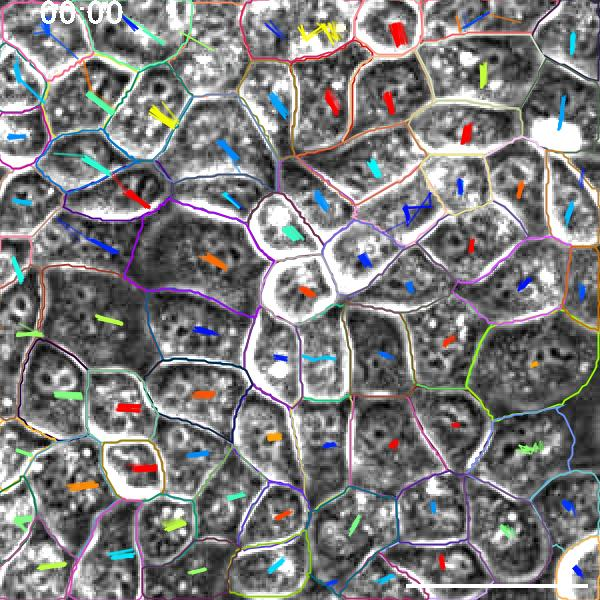
](https://doi.org/10.24355/dbbs.084-202410280821-0)

**Movie S7.** Phase contrast image, segmentation and tracking of radially stretched epithelial cells in monolayer. Segmentation is shown with the contour of each cell in different color. Superimposed cell tracking indicates how cells move during stretch. The scale bar corresponds to 50 μm. Time-lapse movie showing the phase contrast image of MDCK cells subject to 5% radial cyclic stretch. As above, period of stretching is 20 s and the frame interval displayed is 30 s. Superimposed contours in different colors show the cell outlines detected using Cellpose detector of ImageJ plugin Trackmate. Tracks of each cell are also shown and color coded depending on track signature. On the upper left corner, time is indicated in min.
